# Molecular and morphological differentiation of Secret Toad-headed agama, *Phrynocephalus mystaceus*, with the description of a new subspecies from Iran (Reptilia, Agamidae)

**DOI:** 10.3897/zookeys.748.20507

**Published:** 2018-04-05

**Authors:** Evgeniya N. Solovyeva, Evgeniy N. Dunayev, Roman A. Nazarov, Mehdi Radjabizadeh, Nikolay A. Poyarkov, Jr.

**Affiliations:** 1 Zoological Museum of the Lomonosov Moscow State University, Bolshaya Nikitskaya st. 2, Moscow 125009, Russia; 2 Department of Biodiversity, Institute of Environmental Science, International Center for Science, High Technology and Environmental Science, Kerman, Iran; 3 Department of Vertebrate Zoology, Biological Faculty, Lomonosov Moscow State University, Leninskiye Gory, GSP–1, Moscow 119991, Russia

**Keywords:** Khorasan, molecular phylogenetics, morphology, phylogeography, *Phrynocephalus mystaceus khorasanus*, taxonomy

## Abstract

The morphological and genetic variation of a wide-ranging Secret Toad-headed agama, *Phrynocephalus mystaceus* that inhabits sand deserts of south-eastern Europe, Middle East, Middle Asia, and western China is reviewed. Based on the morphological differences and high divergence in COI (mtDNA) gene sequences a new subspecies of *Ph. mystaceus* is described from Khorasan Razavi Province in Iran. Partial sequences of COI mtDNA gene of 31 specimens of *Ph. mystaceus* from 17 localities from all major parts of species range were analyzed. Genetic distances show a deep divergence between *Ph. mystaceus khorasanus* **ssp. n.** from Khorasan Razavi Province and all other populations of *Ph. mystaceus*. The new subspecies can be distinguished from other populations of *Ph. mystaceus* by a combination of several morphological features. Molecular and morphological analyses do not support the validity of other *Ph. mystaceus* subspecies described from Middle Asia and Caspian basin. Geographic variations in the *Ph. mystaceus* species complex and the status of previously described subspecies were discussed.

## Introduction

**Figure 1.**
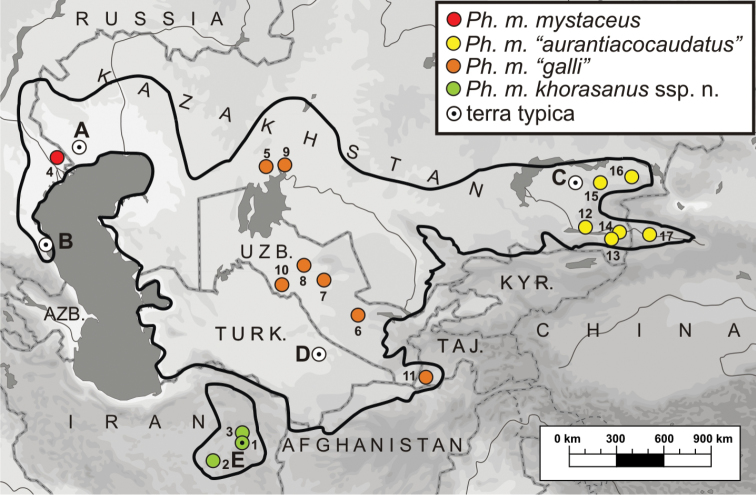
Geographical distribution of *Phrynocephalus mystaceus* and locations of the sites where the samples that were examined in the molecular analyses of the present study were obtained. Locality numbers correspond to those given in Table 1. Dot in the center of a circle indicates the type locality; type localities for taxa are shown as follows: **A** *Lacerta mystacea* Pallas, 1776 **B** *Megalochilus mystaceus dagestanica* Ananjeva, “1986” 1987 **C** *Phrynocephalus mystaceus aurantiacocaudatus* Semenov & Shenbrot, 1990 **D** *Phrynocephalus mystaceus galli* Krassowsky, 1932; and **E** *Ph. mystaceus khorasanus* ssp. n.

Toad-headed agamas of the genus *Phrynocephalus* Kaup, 1825, are distributed from south-eastern Europe and southwest Asia (including the Middle East and Arabian Peninsula) through Middle Asia to Central Asia (northern and central China and Mongolia). This taxonomically complicated genus currently contains up to 32 species (Uetz and Hošek 2016). The secret toad-headed agama, or *Phrynocephalus mystaceus* (Pallas, 1776), is one of the largest representatives of the genus, and is easily distinguished from all other congeners by a pair of large fringed cutaneous folds at the mouth angles. It is a specialized psammophilous species that inhabits sand dunes from Caspian region of the south-eastern part of European Russia in the west to the Ili valley in eastern Kazakhstan and western China in the east, and from Kazakhstan in the north through Middle Asia to northeastern Iran in the south (Bannikov et al. 1977; Zhao and Adler 1993; Anderson 1999; Ananjeva et al. 2004; Molavi et al. 2014; Fig. 1).

*Phrynocephalus mystaceus* was shown to have a high level of anatomical variability (Ananjeva “1986” 1987), which, together with its unique karyotype (Zeng et al. 1997), has led to its uncertain taxonomic classification at the generic level. Eichwald (1831) proposed the new generic name *Megalochilus* Eichwald, 1831 for *Ph. mystaceus*, which was synonymized with the genus *Saccostoma* by Fitzinger (1843). Ananjeva (“1986” 1987) restored the monotypic genus *Megalochilus*, but such taxonomic change was contradicted by Golubev and Sattorov (1992), as they argued that the differences proposed by Ananjeva were too slight to warrant a separate genus status. Molecular phylogenetic analyses based on mtDNA markers failed to resolve the phylogenetic position of *Ph. mystaceus* (Pang et al. 2003), which led Barabanov and Ananjeva (2007) to consider *Megalochilus* as a junior synonym of *Phrynocephalus*. However, a recent phylogeny based on the analysis of *RAG1* nuDNA gene indicated *Ph. mystaceus* as a sister lineage with respect to all other examined *Phrynocephalus* species (Melville et al. 2009). Further study with better taxon sampling based on mtDNA data suggested that *Ph. mystaceus* is a member of the “core” *Phrynocephalus* clade and is associated with *Ph. axillaris* (Solovyeva et al. 2014). The most recent study proposed to consider *Megalochilus* as a subgenus of the genus *Phrynocephalus* (Solovyeva et al. 2014).

There was little consensus in the understanding of intraspecific taxonomy of *Ph. mystaceus*. Krassowky (1932) was the first to split *Ph. mystaceus* into two subspecies: European nominative subspecies *Ph. m. mystaceus* (Pallas, 1776) and Middle-Asian subspecies *Ph. m. galli* Krassowsky, 1932. This taxonomic classification was supported by subsequent studies of Soviet herpetologists (Shibanov 1941; Terentjev and Chernov 1949; Khonyakina 1961). However, morphometric studies by Vel’dre (1964a, 1964b) suggested that it is impossible to distinguish geographical races within *Ph. mystaceus* due to its high morphological variability among populations. Consequently, Ananjeva (1987 “1986”) suggested to upgrade the Middle-Asian subspecies *Ph. m. galli* to full species status and recognized a distinct subspeciesin Daghestan (*Megalochilus mystaceus dagestanica* in Ananjeva et al. 1987 “1986”). Semenov and Shenbrot (1990) analyzed morphological and chromatic differentiation of *Ph. mystaceus* from “Semirechye” (an area east of lake Balkhash in Eastern Kazakhstan), and suggested that this area is inhabited by a distinct subspecies, *Ph. mystaceus aurantiacocaudatus* Semenov et Shenbrot, 1990, which differs from the Middle Asian subspecies *Ph. m. galli* by its bright orange-red coloration of the ventral surface of the tail in young specimens (*versus* lemon-yellow coloration in other subspecies). However, *Ph. mystaceus aurantiacocaudatus* was synonymized with *Ph. m. galli* by Barabanov and Ananjeva (2007) without any discussion.

In summary, three subspecies of *Phrynocephalus mystaceus* are recognized in recent literature (see Barabanov and Ananjeva 2007):

1. *Ph. m. mystaceus* (Pallas, 1776), that inhabits eastern Ciscaucasia (eastern part of Chechen Republic, Daghestan, Kalmykia), Caspian region (southern part of Astrakhan Region, east of the Volga-Ural Sands; introduced to the Apsheron Peninsula, Azerbaijan) and northwestern Kazakhstan (Ananjeva et al. 2004). **Terra typica restricta**: Ryn-Peski (Ryn Sands), Ural Region, northwestern Kazakhstan (Barabanov and Ananjeva 2007). This form includes *Megalochilus mystaceus dagestanica* Ananjeva, “1986” 1987, described from Kumtorkala, Daghestan, Russia, as a junior synonym.

2. *Ph. mystaceus galli* Krassowsky, 1932, that inhabits Transcaspian Region and Middle Asia from Turkmenistan, Uzbekistan, Kazakhstan, to northeastern and eastern Iran and adjacent areas of Afghanistan (Anderson 1999; Ananjeva et al. 2004). **Terra typica**: Repetek station, Lebapsky District, Turkmenistan (Barabanov and Ananjeva 2007). Based on its distribution, this subspecies is supposed to inhabit north-eastern Iran (Anderson 1999).

3. *Ph. mystaceus aurantiacocaudatus* Semenov & Shenbrot, 1990, known from eastern Kazakhstan and western China (Ili River Valley in Xinjiang). **Terra typica**: 70 km north northwest of Ushtobe, Eastern Kazakhstan. Regarded as a junior synonym of *Ph. mystaceus galli* by Barabanov and Ananjeva (2007), however, without any justification.

It is notable that all previous works on geographic variations of *Ph. mystaceus* omitted populations from the southernmost edge of its range, Iran and Afghanistan, from the analyses. Morphological characterization and analysis of distribution of *Ph. mystaceus* in Iran was carried out by Anderson (1999) and Molavi et al. (2014). Anderson (1999) examined specimens from Iran and Uzbekistan, and proposed that Iranian populations demonstrate intermediate morphology between *Ph. m. galli* and *Ph. mystaceus*. Molavi et al. (2014), based on a study of seven specimens from Semnan Province, repeated earlier conclusions by Anderson (1999) and suggested that further investigation of both morphological and molecular characters are required to clarify the taxonomic status of Iranian *Ph. mystaceus* populations.

The recent analysis of phylogenetic relationships within the genus *Phrynocephalus* based on four mitochondrial genes revealed a remarkable divergence between *Ph. mystaceus* samples from Iran and Middle Asia (Solovyeva et al. 2014). Based on these results the Iranian population was tentatively indicated as a putative new subspecies *Ph. mystaceus* ssp. In the present study, we provide a detailed analysis of both morphological and genetic variation of *Ph. mystaceus* across its range and confirm deep differentiation between the population from Khorasan Province of Iran and other populations in the species range. The currently recognized subspecies of *Ph. mystaceus* are reviewed and a new subspecies from Khorasan Province is described, based on both molecular and morphological features.

## Materials and methods

**Sampling.** Historical collections of the Zoological Museum of Lomonosov Moscow State University (**ZMMU**) were examined, in total, 70 adult and subadult specimens of all currently recognized subspecies (Appendix 1). In addition, type specimens of *Ph. mystaceus galli* (lectotype, ZMMU R-6413) and *Ph. mystaceus aurantiacocaudatus* (holotype, ZMMU R-6412) were also examined. Sampling was carried out in the Khorasan Province of Iran in April of 2005, April of 2006, May and June of 2009, and May of 2010. Specimens from Iran were obtained through the collaboration with the Zoological Museum of International Center for Science, High Technology and Environmental Sciences (**ICSTZM**; Kerman, Iran; MOU no. 158/2010). Tissue samples from 31 *Ph. mystaceus* specimens were used in molecular analyses, and their geographic distribution is shown in Fig. 1. Details on museum IDs and localities of origin for each sample are summarized in Table 1.

*Molecular analyses*. Mitochondrial DNA COI gene (cytochrome oxidase *c* subunit I) fragment, 654 b. p. in length was analyzed. Muscle and skin tissues were disintegrated with Proteinase K and total genomic DNA was extracted using a standard phenol-chloroform extraction protocol followed by ethanol precipitation of DNA (Sambrook et al. 1989). PCR amplification was performed using MyCycler BioRad under conditions described by Ivanova et al. (2006). Standard pair of primers was used: VF1d (5'-TTCTCAACCAACCACAARGAYATYGG-3') and VR1d (5'-TAGACTTCTGGGTGGCCRAARAAYCA-3') or Rep-COI-F (5'-TNTTMTCAACNAACCACAAAGA-3') and Rep-COI-R (5'-ACTTCTGGRTGKCCAAARAATCA-3'). PCR reaction volume was 20 µl and it contained ca. 100 ng of template DNA, 0.3 pM/µl of each PCR primer, 1xTaq-buffer with 25 mM of MgCl_2_ (Silex, Moscow Russia), 0.2 mM dNTPs, and 1 unit of Taq-polymerase (Silex, Moscow Russia; 5 units/µl). The results of the amplification were examined using electrophoresis in 1% agarose gel in presence of ethidium bromide. The length of the obtained fragments was 680 bp. We included two sequences of *Ph. mystaceus* from western China available from Genbank (NC022131 and KC578685; see Chen et al. 2014) in the analyses. Samples of *Ph. melanurus* (ZMMU R-12328, GenBank AN MF567976) and *Trapelus sanguinolentus* (ZMMU R-12709, GenBank AN KF691668) were used as outgroups.

Sequences were aligned using Seqman 5.06 and checked using BioEdit Sequence Alignment Editor 7.1.3.0 (Hall 1999). All sequences were deposited in GenBank (see Table 1 for all voucher information, with corresponding GenBank accession numbers).

**Table 1.** List of the samples used in molecular analyses. Locality numbers correspond to those in Figure 1.

Voucher N^o^	Subspecies	Locality	GenBank N^o^
ZMMU R-12202	*Ph. mystaceus khorasanus* ssp. n.	Iran, Khorasan Razavi Prov., Gonabad **(1)**	MF567983
ZMMU R-13009-1	*Ph. mystaceus khorasanus* ssp. n.	Iran, Khorasan Razavi Prov., Boshrue **(2)**	MF567989
ZMMU R-13009-2	*Ph. mystaceus khorasanus* ssp. n.	Iran, Khorasan Razavi Prov., Boshrue **(2)**	KF691714
ZMMU R-13011-1	*Ph. mystaceus khorasanus* ssp. n.	Iran, Khorasan Razavi Prov., Gonabad **(1)**	MF567987
ZMMU R-13011-2	*Ph. mystaceus khorasanus* ssp. n.	Iran, Khorasan Razavi Prov., Gonabad **(1)**	MF567988
ZMMU R-11913	*Ph. mystaceus khorasanus* ssp. n.	Iran, Khorasan Razavi Prov., Gonabad **(1)**	MF567975
ZMMU R-13169	*Ph. mystaceus khorasanus* ssp. n.	Iran, Khorasan Razavi Prov., 30 km N Gonabad **(3)**	MF567974
RuHF-072-1	*Ph. mystaceus mystaceus*	Russia, Astrakhan Prov., Dosang **(4)**	MF567968
RuHF-072-2	*Ph. mystaceus mystaceus*	Russia, Astrakhan Prov., Dosang **(4)**	MF567969
ZMMU-R-12457-2	*Ph. mystaceus mystaceus*	Russia, Astrakhan Prov., Dosang **(4)**	MF567990
ZMMU-R-12457-3	*Ph. mystaceus mystaceus*	Russia, Astrakhan Prov., Dosang **(4)**	MF567986
RuHF-079-1	*Ph. mystaceus galli*	Kazakhstan, N Priaralye, S border of Malye Barsuki sands **(5)**	MF567971
RuHF-079-2	*Ph. mystaceus galli*	Kazakhstan, N Priaralye, S border of Malye Barsuki sands **(5)**	MF567970
ZMMU-R-12517-2	*Ph. mystaceus galli*	Kazakhstan, N Priaralye, S border of Malye Barsuki sands **(5)**	MF567985
ZMMU R-12772	*Ph. mystaceus galli*	Kazakhstan, Aralsk **(6)**	MF567982
ZMMU R-12775	*Ph. mystaceus galli*	Uzbekistan, Qarakalpaqiston Republic **(7)**	MF567981
ZMMU-R-12266	*Ph. mystaceus galli*	Uzbekistan, Qarakalpaqiston Republic, Chukurkak **(8)**	MF567978
ZMMU-R-12252-1	*Ph. mystaceus galli*	Uzbekistan, Navoi Prov., Terankuduk **(9)**	MF567977
ZMMU-R-12261-1	*Ph. mystaceus galli*	Uzbekistan, Navoi Prov., Yamankum desert **(10)**	KF691713
ZMMU R-12799	*Ph. mystaceus galli*	Tajikistan, Shaartuz **(11)**	MF567979
RuHF-077-1	*Ph. mystaceus aurantiacocaudatus*	E Kazakhstan, N Kapchagai Reservoir **(12)**	MF567972
RuHF-077-2	*Ph. mystaceus aurantiacocaudatus*	E Kazakhstan, N Kapchagai Reservoir **(12)**	MF567973
ZMMU R-12518	*Ph. mystaceus aurantiacocaudatus*	SE Kazakhstan, left bank of Ili River,125 km of the road Almaty-Bakanas **(13)**	MF567984
ZMMU R-12778	*Ph. mystaceus aurantiacocaudatus*	Kazakhstan, Pidzhim env. **(14)**	MF567980
ZMMU R-14715-1	*Ph. mystaceus aurantiacocaudatus*	Kazakhstan, S Balkhash lake, N of Matay **(15)**	MF567991
ZMMU R-14715-2	*Ph. mystaceus aurantiacocaudatus*	Kazakhstan, S Balkhash lake, N of Matay **(15)**	MF567992
ZMMU R-14715-3	*Ph. mystaceus aurantiacocaudatus*	Kazakhstan, S Balkhash lake, N of Matay **(15)**	MF567993
ZMMU R-14715-4	*Ph. mystaceus aurantiacocaudatus*	Kazakhstan, S Balkhash lake, N of Matay **(15)**	MF567994
ZMMU NAP-05510	*Ph. mystaceus aurantiacocaudatus*	Kazakhstan, E Balkhash lake, environs of Kabanbay **(16)**	MF567995
No voucher number	*Ph. mystaceus aurantiacocaudatus*	China, Ili River valley, Huocheng **(17)**	NC021131

Mean uncorrected *p*-distances and sequences characteristics were calculated using MEGA 6 (Tamura et al. 2011). Phylogenetic analyses were conducted using Treefinder (Jobb et al. 2011) and MrBayes 3.1.2 (Huelsenbeck and Ronquist 2001; Ronquist and Huelsenbeck 2003) software.

PartitionFinder v1.0.1 (Lanfear et al. 2012) was used to estimate the optimal evolutionary models for Bayesian inference analysis. The preferred model for *COI* alignment was HKY + G for two partitions (codon position 1 and 2 vs. codon position 3) as suggested by the Akaike information criterion (AIC). Bayesian phylogenetic analysis was performed using MrBayes v.3.1.2 (Ronquist and Huelsenbeck 2003) with two simultaneous runs, each with four chains, for 20 million generations, 2 million generations were cut as burn in. The convergence of the runs was checked to make sure that the effective sample sizes (ESS) were all above 200 by examining the likelihood plots using TRACER v.1.5 (Rambaut and Drummond 2007).

The Maximum Likelihood (ML) analysis was conducted using Treefinder (Jobb et al. 2011). Each dataset was divided into three partitions according to codon positions; for each partition the best fitting substitution model was selected using the AIC in Treefinder. For ML-analysis we used 1000 pseudoreplics (BS) and Expected Likelihood Weights (ELW).

Confidence in tree topology was tested by using non-parametric bootstrap analysis (Felsenstein 1985) with 1000 replicates and posterior probability (PP) for Bayesian inference (BA) in MrBayes 3.1.2 (Huelsenbeck and Ronquist 2001). Branches with bootstrap values of 70% or higher and posterior probabilities values over 0.95 were regarded as sufficiently resolved (Huelsenbeck and Hillis 1993).

*Morphological analyses*. Pholidosis was examined and morphometrics acquired for 79 individuals in four groups of *Ph. mystaceus*, including 20 specimens of nominative subspecies *Ph. m. mystaceus*, seven specimens from Khorasan Province of Iran, 32 specimens of *Ph. m. aurantiacocaudatus* from Eastern Kazakhstan, and 20 specimens of *Ph. m. galli* from Middle Asia (Appendix 1). In order to take into account sexual dimorphism, males (n = 26) and females (n = 44) were analyzed separately.

*Morphological characteristics* and the methods for their measurement are generally the same as in the study by Solovyeva et al. (2012). The following measurements and scalation counts were used: (1) snout-vent length (SVL); (2) tail length (TL); (3) SVL/TL ratio; (4) number of flat supralabials anterior to angular enlarged spine-like supralabial scales (SLbA); (5) total number of flat supralabials from tip of snout to insertion of cutaneous fold at mouth angle (SL); (6) relative length of the dark distal part of the tail to the total tail length (in ventral aspect, calculated as TL-black/TL ratio); (7) number of scales surrounding subnasal from below (SSbNb); (8) subnasal in contact with medial side of supranasal (*vs.* subnasal not in contact with medial side of supranasal) (SbN-SpN); (9) supranasal edges nostril dorsally along the full length of nostril (*vs.* supranasal edges nostril dorsally along only half of nostril length) (SpN); (10) height of supranasal is less than or equal to height of subnasal (*vs.* height of supranasal exceeds height of subnasal) (hSpN SbN); (11) number of scale rows that separate subnasal and labial scales (SbN-L); (12) longitudinal row of white scales in supraorbital area outlined by continuous black lines (or intermitted) (WS&BL); (13) number of small rows of scales between anterior (2d and 3d) inframandibulars and large rows of scales under infralabial scales – 1-2 or 2-3 (aIMd-IL); (14) number of scales that underlay enlarged spiny scales on edge of cutaneous fold at mouth angle (SuSSCF); (15) number of small granular scales between posteriormost supralabial and insertion of cutaneous fold at mouth angle (pSL-CF); (16) number of flat infralabials anterior to angular enlarged spine-like infralabial scales (ILbA); (17) total number of infralabials from tip of snout to insertion of cutaneous fold at mouth angle (IL); (18) number of subdigital lamellae under toe III (SLIII); (19) number of enlarged triangular scales on lateral fringes of toe III (FrIII); (20) number of subdigital lamellae under toe IV (SLIV); (21) number of enlarged triangular scales on lateral fringes of toe IV (FrIV). Characteristics 18-21 (SLIII, FrIII, SLIV, FrIV) were registered with no regard to the sex of the individual. Following standard measurements were additionally taken for holotype and paratypes: head height (HH); head length (HL, measured on ventral side from snout tip to gular fold); head width (HW, measured at broadest part of head excluding cutaneous folds); pileus width (PW). Measurements were taken using a digital caliper and rounded to the nearest 0.1 mm.

Box-and-whiskers-plots and values of descriptive statistics were calculated using R (R Core Team, 2013). The Mann-Whitney test of independent series was used to determine the differences between the pairs of subspecies (with confidence level of p ≤ 0.05). Principal Components Analysis (PCA) was performed using R (R Core Team, 2013) to visualize morphological variation between Khorasan specimens of *Ph. mystaceus* and specimens from other populations.

## Results

**Sequence characteristics.** The sequenced fragments from 31 *Ph. mystaceus* specimens were up to 654 b.p. in length, among which 577 sites were identified as conservative, 74 as variable and 59 as potentially parsimony-informative. Nucleotide frequencies were equal to: 30.2% (A), 27.5% (T/U), 27.9% (C), and 14.4% (G). The transition-transversion bias (R) was estimated to be 6.574 (all data given for in-group only).

**Figure 2.**
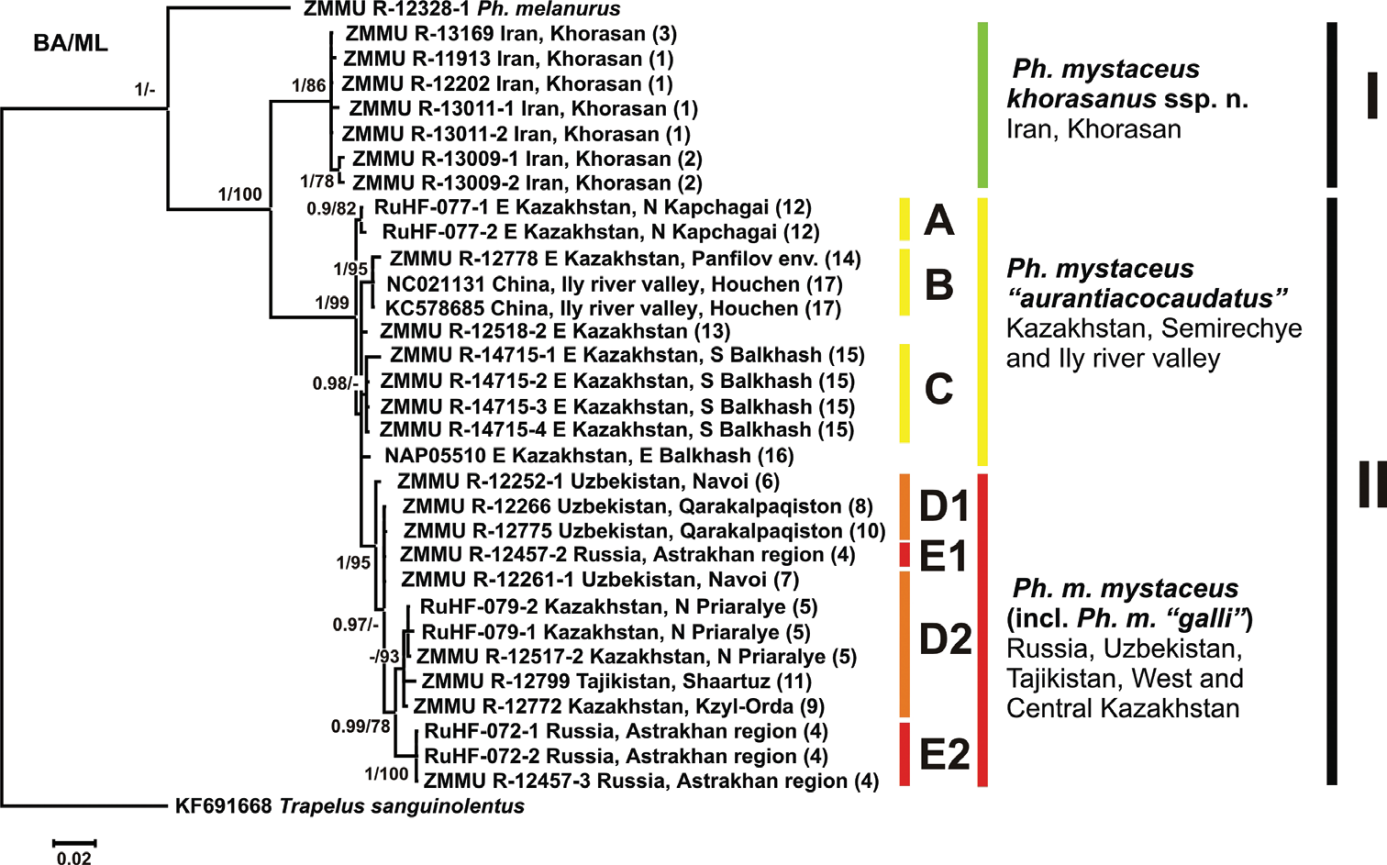
BI-inferred dendrogram that illustrates the phylogenetic relationships of the *Phrynocephalus mystaceus* species complex based on the analysis of 654 b. p. fragment of *COI* gene (mtDNA). Numbers at the tree nodes show Bayesian Posterior Probabilities/ Maximum Likelihood Bootstrap Support. Only PP values higher than 0.90 and BS values higher than 75% are shown. *COI* sequence of *Trapelus sanguinolentus* is used as an outgroup.

**Phylogenetic analysis.** The results of phylogenetic analysis are presented in Fig. 2. BI and ML yielded trees that show essentially similar topologies. All analyses reveal the presence of two reciprocally monophyletic clades within *Ph. mystaceus*. The first clade consists of Irainan *Ph. mystaceus* ssp. from Khorasan Province (node support values are 1.0/86; hereafter given for BI PP/ ML BS; clade I on Fig. 2). The second clade includes all other *Ph. mystaceus* populations from Middle and Central Asia and Caspian Region (1.0/99; clade II on Fig. 2). Further phylogenetic structure within the second clade of non-Iranian *Ph. mystaceus* is poorly resolved. Populations from the eastern part of the range including Eastern Kazakhstan and Xinjiang (China) that correspond to the *Ph. m.* “*aurantiacocaudatus*” occupy basal position in the clade II, but are not monophyletic and fall into three poorly differentiated subclades: from the environs of Kapchagai (subclade A; 0.90/82), Ili River Valley (subclade B; from Zharkent to Xinjiang; 1.0/95), and the environs of Lake Balkhash (subclade C; 0.98/-) (see Fig. 2). Phylogenetic positions of two samples from Eastern Kazakhstan (ZMMU NAP-05510 and ZMMU R-12518-2) are not resolved. All other populations from Middle Asia (Kazakhstan and Uzbekistan – Fig. 2D) and Caspian Region (Astrakhan Province, Russia – Fig. 2E) form a significantly monophyletic clade (1.0/95), which is deeply nested within the basal differentiation of East Kazakhstan *Ph. m.* “*aurantiacocaudatus*” clades (see Fig. 2), rendering the latter taxon paraphyletic. The Middle Asian – Caspian clade (D + E) corresponds to the nominative subspecies *Ph. mystaceus mystaceus* and also includes populations previously classified as *Ph. mystaceus galli* (Aral Sea Region, Uzbekistan and Tajikistan – Fig. 2D). Populations of *Ph. mystaceus mystaceus* and *Ph. mystaceus* “*galli*” are mixed with each other without any clear structure (see Fig. 2 D1, D2, E1, E2).

**Genetic distances.** Uncorrected genetic *p*-distances between and within clades of *Ph. mystaceus* are shown in Table 2. The *p*-distances within the Middle Asian – Caspian clade of *Ph. mystaceus*, including comparisons between different lineages of *Ph. m.* “*aurantiacocaudatus*” and between *Ph. m.* “*aurantiacocaudatus*” and *Ph. m. mystaceus* are quite low (0.55–0.88% and 1.56–1.87%, respectively), which is less than intraspecific genetic distances for *COI* for some other species of *Phrynocephalus* (e.g. see Solovyeva et al. 2011 for *Ph. helioscopus*). However, *p*-distances between *Ph. mystaceus* ssp. from Khorasan Province and all other groups of *Ph. mystaceus* are very high (6.84–7.28%), they even exceed interspecific genetic distances for *COI* gene reported for certain species of *Phrynocephalus* (Solovyeva et al. 2014). This data clearly suggest a deep divergence between *Ph. mystaceus* populations from Khorasan Province and populations from the rest of the range of the species.

**Table 2.** Uncorrected *p*-distances (percentage) between and within the groups of *Ph. mystaceus* complex. Distances are shown under the diagonal row; standard error values are given above the diagonal row. *Ph. m. aurantiacocaudatus* A corresponds to the population from N Kapchagai (RuHF-077); a – all specimens of *Ph. m. aurantiacocaudatus*, except for *Ph. m. aurantiacocaudatus* A, b – *Ph. m. aurantiacocaudatus* from southeast Pribalkhashye (Matay) (ZMMU R-14715), c – *Ph. m. aurantiacocaudatus* from Ili river valley, except for R-12518-2.

Group		1	2			3	4
			2-a	2-b	2-c		
**1. *Ph. m. mystaceus* [Including “*Ph. m. galli*”)**		**0.97**	0.4	0.47	0.43	0.41	0.96
**2. *Ph. m.* “*aurantiacocaudatus***”	**2-a**	1.7	**0.63**	–	–	0.22	0.94
	**2-b**	1.87	–	**0.16**	0.34	0.32	1.02
	**2-c**	1.65	–	0.88	**0.27**	0.25	1.03
**3. *Ph. m.* “*aurantiacocaudatus*” A**		1.56	0.66	0.88	0.55	**0.15**	0.97
**4. *Ph. m. khorasanus* ssp. n.**		7.28	7.18	7.24	7.17	6.84	**0.37**

**Figure 3.**
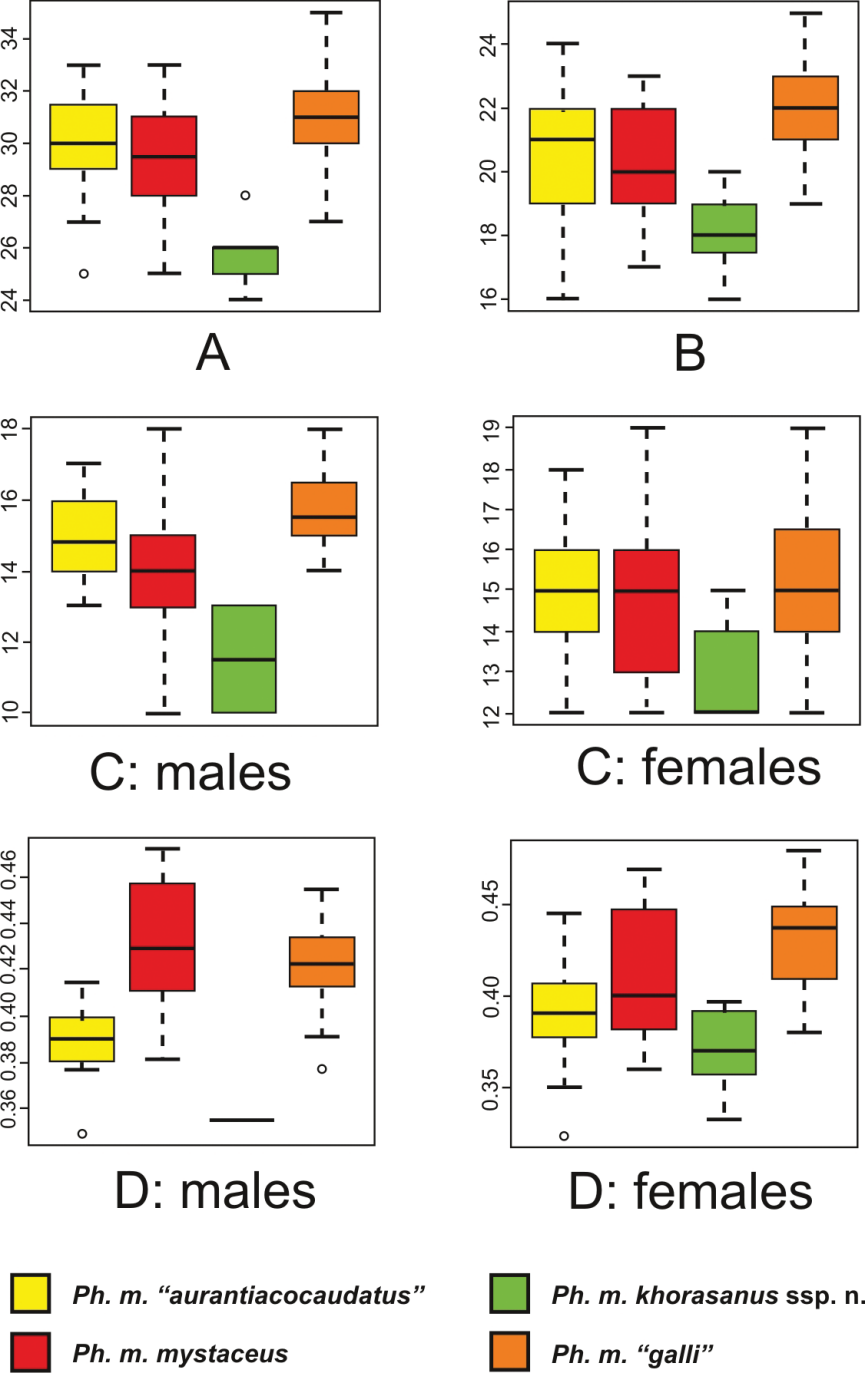
Statistically significant morphological differences between *Ph. mystaceus khorasanus* ssp. from Iran and other subspecies of *Ph. mystaceus*: **A** the number of subdigital lamellae on the toe IV (SLIV) **B** the number of enlarged triangular scales on the lateral fringe of the toe III (FrIII) **C** the total number of supralabial scales (SL) **D** the relative length of the dark distal part of the tail to the total tail length (TL-black/TL).

**Morphology**. Our study supports the results of previous researchers that indicated very high morphological variation in the absence of consistent morphological variation patterns that could delimit recognized subspecies in Middle Asian populations of *Ph. mystaceus* (Vel’dre 1964a, 1964b; Semenov and Shenbrot 1990). Most characteristics, including body size, were uninformative for distinguishing subspecies and local populations of *Ph. mystaceus*. Only four morphological characteristics showed consistent differences between Iranian and Middle Asian/Caspian populations of *Ph. mystaceus*, including SLIV, FrIII, SL, and TL-black/TL. Specifically, SLIV was lower in the population from Khorasan Razavi Province (N = 7) than in other subspecies of *Ph. mystaceus* (differences are significant; p = 0.000 for comparison with *Ph. h.* “*aurantiacocaudatus*”, N = 32; p = 0.000 for comparison with *Ph. h.* “*galli*”, N = 20; p = 0.087 for comparison with *Ph. m. mystaceus* sensu stricto, N = 20; for measurement ranges see Table 3). FrIII was also significantly lower in Khorasan population (N = 7) than in *Ph. m. mystaceus* sensu stricto (p = 0.000 N = 20). SL was also lower in Khorasan population (N = 7) than in other subspecies (p = 0.007 for comparison with *Ph. m.* “*aurantiacocaudatus*”, N = 32; p = 0.001 for comparison with *Ph. m. mystaceus* sensu stricto, N = 20; p = 0.050 for comparison with *Ph. m.* “*galli*”, N = 20). Finally, the dark distal part of the tail (TL-black/TL) was relatively longer in the Khorasan population (differences are significant; p = 0.023, for comparison with *Ph. m.* “*aurantiacocaudatus*”, N = 32; p = 0.000 for comparison with *Ph. m. mystaceus* sensu stricto, N = 20; p = 0.001 for comparison with *Ph. m.* “*galli*”, N = 20). Morphological comparison of four geographical population groups that correspond to the subspecies “*mystaceus* sensu stricto”, “*galli*”, “*aurantiacocaudatus*” and the “Khorasan population” for the diagnostic morphological characteristics mentioned above is given in Fig. 3. Other characteristics with p-values for pairwise comparisons <0.05 showed significant overlap of values between subspecies and cannot be reliably used in diagnostics; p-values for morphological characteristics for pairwise comparisons are summarized in Appendix 2. Standard measurements of *Ph. mystaceus* ssp. from Khorasan Province are presented in Table 4.

**Figure 4.**
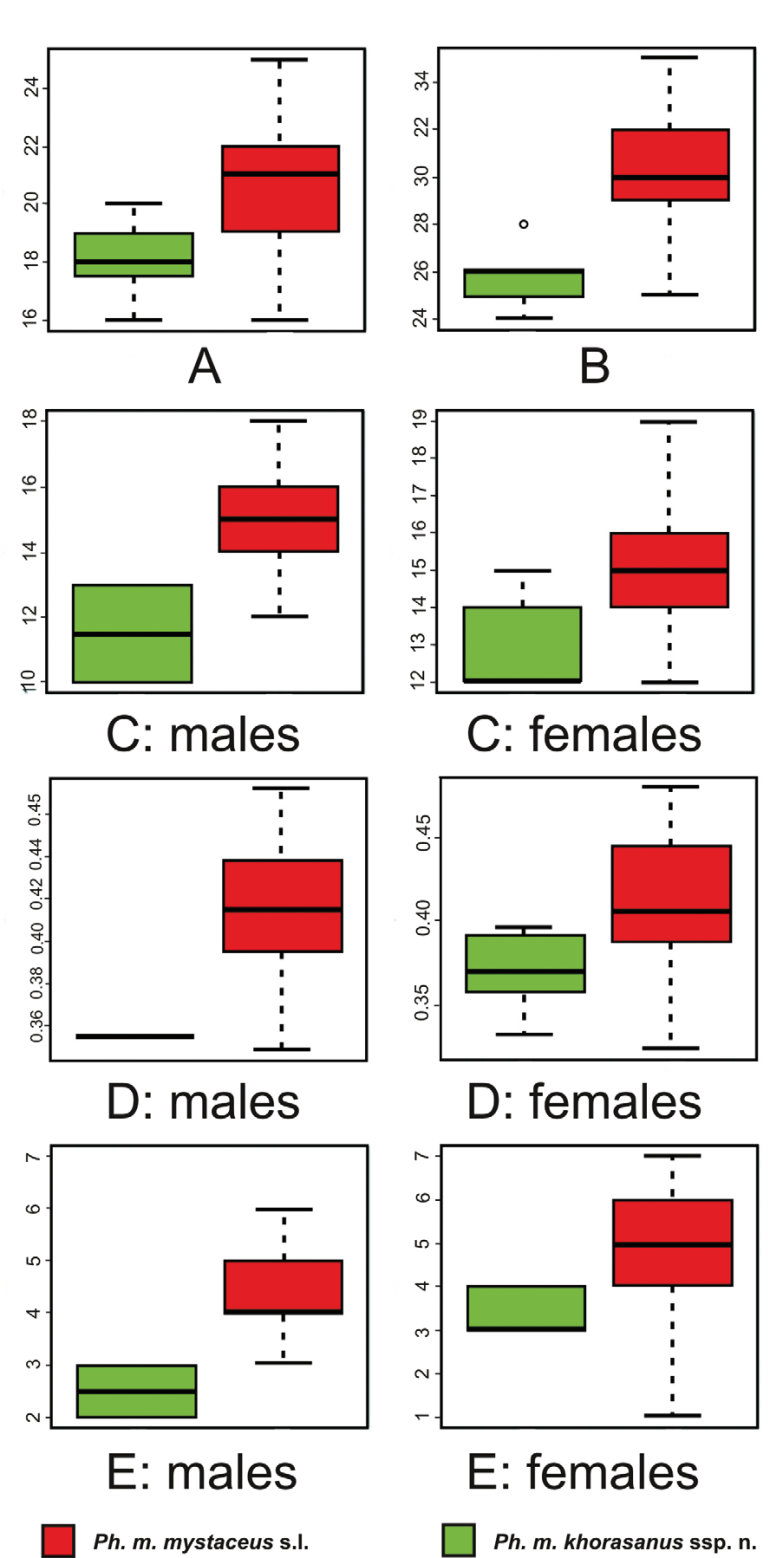
Statistically significant morphological differences between *Ph. mystaceus khorasanus* ssp. from Iran and other *Ph. mystaceus*: **A** the number of subdigital lamellae on the toe III (SLIII) **B** the number of subdigital lamellae on the toe IV (SLIV) **C** the total number of supralabial scales (SL) **D** the relative length of the dark distal part of the tail to the total tail length (TL-black/TL) **E** number of flat infralabials anterior to the angular enlarged spine-like infralabial scales (IlbA).

Comparison of Khorasan *Ph. mystaceus* ssp. population with other populations of *Ph. mystaceus* from Middle Asia and Caspian region (data for “*mystaceus* sensu stricto”, “*galli*”, “*aurantiacocaudatus*” combined together) also demonstrated significant differences for many traits with p<0.05 (Appendix 2), however, values for most of them were overlapping. The six of the characters with the minimal overlap were the following: SVL/TL, TL-black/TL, SL, ILbA, SLIII, SLIV (see Fig. 4 for details).

**Figure 5.**
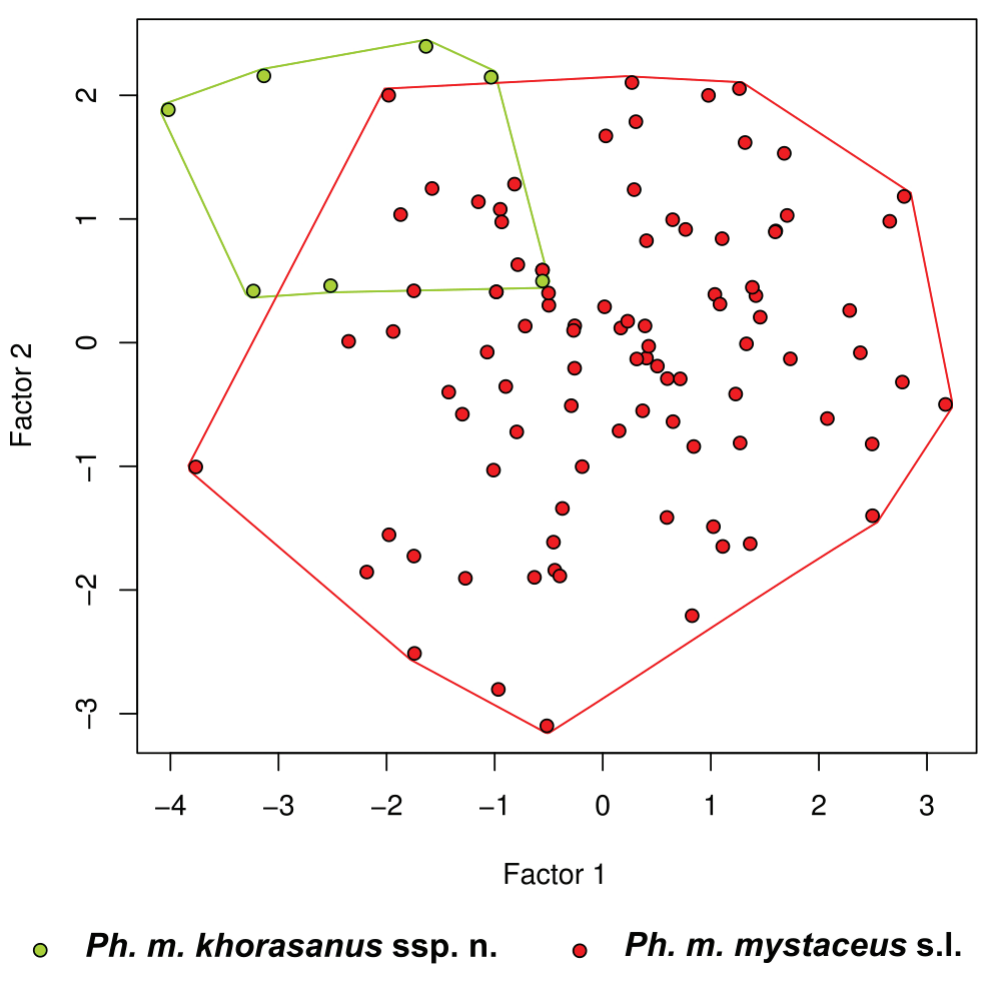
Principal Components Analysis (PCA) of 19 morphological traits (excluding SVL and TL).

PCA showed differences between Khorasan population and other *Ph. mystaceus* populations, although these two groups are slightly overlapping with two Khorasan specimens falling into the *Ph. m. mystaceus* sensu lato area (Fig. 5). PCA failed to reveal any clear structuring within the Middle Asian / Caspian populations of *Ph. mystaceus*.

**Table 3.** Mean, standard deviation, and range of measurements (mm) of adult *Ph. mystaceus* ssp. For abbreviations, see Materials and methods. In SbN-SpN, SpN, hSpN-SbN, and WS&BL: 0 equals “no”, 1 equals “yes”.

Subspecies	A. *mystaceus* s. str.		B. ** “*galli*”		C. ** “*aurantiacocaudatus*”		D. *mystaceus* s. l. (A+B+C)		E. *khorasanus* ssp. n.	
**Measure-ments**	**m** **N = 9**	**f** **N = 11**	**m** **N = 7**	**f** **N = 13**	**m** **N = 8**	**f** **N = 15**	**m** **N = 26**	**f** **N = 44**	**m** **N = 2**	**f** **N = 5**
**SVL**	10.13±1.06 (7.9–11.2)	9.26±0.89 (6.3–10.5)	8.41±1.00 (6.4–9.5)	8.18±1.02 (6.3–10)	8.10±1.23 (6.5–10.5)	6.97±1.31 (5.5–10)	9.01±1.42 (6.4–11.2)	8.19±1.41 (6.3–10.5)	8.55±0.07 (8.5–8.6)	5.96±0.65 (5.4–7)
**TL**	11.12±0.97 (9.3–11.2)	10.14±0.87 (7.3–11.2)	8.53±0.99 (6.4–9.7)	7.28±1.03 (6.1–10.6)	8.30±1.50 (7–11.5)	7.27±1.28 (6–10.5)	9.50±1.74 (6.4–11.5)	8.49±1.63 (6–11.2)	7.60±0.00 (7.6)	5.8±0.61 (5.1–6.7)
**SVL/TL**	0.92±0.05 (0.85–1.00)	0.91±0.04 (0.85–0.97	0.97±0.05 0.93–1.06	1.02±0.05 (0.83–1.10	0.98±0.04 (0.91–1.03)	0.96±0.04 (0.88–1.03)	0.96±0.05 (0.85–1.06)	0.97±0.07 (0.83–1.10)	1.13±0,01 (1.12–1.13)	1.03±0.03 (1.00–1.06)
**SLbA**	12.11±0.60 (11–13)	11.6±3.03 (7–17)	10.14±1.35 (8–12)	11.25±1.95 (7–15)	10.5±1.77 (8–14)	11±1.63 (7–13)	10.93±1.55 (8–14)	11.06±2.27 (7–17)	8.50±3.53 (6–11)	9.00±1.73 (8–11)
**SL**	15.78±1.39 (14–18)	16.11±1.90 (13–19)	13±1.63 (10–15)	14.75±1.88 (12–19)	14.75±1.49 (13–17)	14.69±1.54 (12–18)	14.83±1.76 (10–18)	14.94±1.77 (12–19)	11.5±2.12 (10–13)	12.67±1.15 (12–14)
**TL-black/TL**	0.42±0.02 (0.38–0.45)	0.44±0.03 (0.39–0.48)	0.44±0.02 (0.41–0.47)	0.41±0.04 (0.33–0.47)	0.39±0.02 (0.35–0.41)	0.39±0.03 (0.32–0.45)	0.42±0.03 (0.35–0.47)	0.41±0.04 (0.32–0.48)	0.36±0.00 (0.36)	0.39±0.01 (0.37–0.40)
**SSbNb**	5±0.71 (4–6)	4.8±0.42 (4–5)	6.14±0.69 (5–7)	5.81±1.38 (3–8)	5.75±1.(8 4–8)	5.13±1.09 (4–7)	5.57±0.94 (4–8)	5.38±1.16 (3–8)	5±1.41 (4–6)	4.33±1.53 (3–6)
**SbN-SpN**	0.44±0.53 (0–1)	0.40±0.52 (0–1)	0.71±0.49 (0–1)	0.56±0.51 (0–1)	0.38±0.52 (0–1)	0.13±0.34 (0–1)	0.43±0.50 (0–1)	0.38±0.49 (0–1)	0.50±0.71 (0–1)	0±0.00 (0)
**SpN**	0.33±0.50 (0–1)	0.50±0.53 (0–1)	0.71±0.49 (0–1)	0.88±0.34 (0–1)	0.25±0.46 (0–1)	0.56±0.51 (0–1)	0.50±0.50 (0–1)	0.63±0.49 (0–1)	0±0.00 (0)	0.67±0.58 (0–1)
**hSpN-SbN**	0.78±0.44 (0–1)	0.90±0.32 (0–1)	0.57±0.53 (0–1)	0.44±0.51 (0–1)	0.88±0.35 (0–1)	0.56±0.51 (0–1)	0.70±0.47 (0–1)	0.65±0.48 (0–1)	0±0.00 (0)	0±0.00 (0)
**SbN-L**	5.22±1.39 (3–8)	5.30±0.79 (4.5–7)	4.14±1.07 (3–6)	4.78±1.22 (3–7	3.88±0.64 3–5)	4.34±0.60 (3–5	4.50±1.14 (3–8)	4.76±1.03 (3–7)	5.00±0.00 (5)	5±0.00 (5)
**WS&BL**	1±0.00 (1)	1±0.00 (1)	1±0.00 (1)	0.94±0.25 (0–1	0.75±0.46 0–1)	0.75±0.41 (0–1)	0.90±0.31 (0–1)	0.88±0.32 (0–1)	0.5±0.71 (0–1)	0.33±0.58 (0–1)
**aIMd-IL**	2±0.87 (1–3)	1.95±1.30 (0–4)	2±0.58 (1–3)	1.59±0.84 (0–3	1.88±0.64 1–3	1.31±0.48 (1–2)	1.80±0.71 (1–3)	1.57±0.83 (0–4)	1.5±0.71 (1–2)	2.33±0.29 (2–2.5)
**SuSSCF**	2.4±0.53 (2–3)	2.60±0.52 (2–3)	1.79±0.49 (1–2.5)	1.72±0.55 (1–3)	2.31±0.70 (1–3)	2.41±0.46 (2–3)	1.98±0.79 (1–3)	2.14±0.63 (1–3)	2±0.71 (1.5–2.5)	1.67±0.58 (1–2)
**pSL-CF**	1.56±0.53 (1–2)	1.60±0.70 (0–2)	2.14±0.90 (1–3)	2.19±0.91 (1–4)	1.25±0.71 (0–2)	1.36±0.5 (1–2)	1.77±0.77 (0–2)	1.83±0.75 (0–4)	1.2±0.71 (1–2)	0.67±0.58 (0–1)
**ILbA**	4.17±1.03 (3–6)	4±1.38 (1–6)	5.10±1.20 (3–7)	5.29±1.07 (2.5–7)	4.5±0.53 (4–5)	5.07±1.62 (2–7)	4.57±1.04 (3–7)	4.70±1.50 (1–7)	2.50±0.71 (2–3)	3.4±0.55 (3–4)
**IL**	6.5±1.45 (3–9)	7.15±1.04 (5–9)	7.50±1.51 (5–10)	7.47±0.92 (6–9)	6.75±0.71 (6–7)	6.6±1.24 (3–8)	6.9±1.35 (3–10)	7.08±1.13 (3–9)	6±1.41 (5–7)	5.2±2.05 (3–7)
**SLIII**	21.77±1.61 (19–25)		20.27±1.68 (17–29)		20.44±2.05 (16–24)		20.79±1.89 (16–29)		18.14±1.45 (16–20)	
**FrIII**	9.97±0.85 (9–12)		9.87±0.97 (8–12)		8.31±0.86 (7–11)		9.35±1.18 (7–12)		8.86±1.35 (7–11)	
**SLIV**	30.93±1.95 (27–35)		29.67±2.11 (25–35)		30.03±1.93 (25–33)		30.15±1.99 (25–35)		25.71±1.25 (24–27)	
**FrIV**	19.97±1.87 (16–25)		18.87±1.70 (16–23)		16±1.87 (13–21)		18.22±2.48 (13–25)		18.86±1.21 (18–21)	

**Table 4.** Measurements (mm) of adult *Ph. mystaceus khorasanus* ssp. n. For abbreviations, see Materials and methods. In SbN-SpN, SpN, hSpN-SbN and WS&BL: 0 equals “no”, 1 equals “yes”.

Measurements	ZMMU Specimen ID	SVL	TL	SVL/TL	HL	HH	HW	PW	SLbA	SL	TL-black/TL	SSbNb	SbN-SpN	ZMMU Specimen ID	SpN	hSpN- SbN	SbN-L	WS&BL	aIMd-IL	SuSSCF	pSL-CF	ILbA	IL	SLIII	FrIII	SLIV	FrIV
**Males (N = 2)**	R-13011-1	85.0	76.0	1.12	19.8	11.0	21.7	13.8	6	10	0.355	6	0	R-13011-1	0	0	5	1	2	1.5	2	3	7	18	10	26	21
	R-13009-1	86.0	76.0	1.13	19.3	11.8	18.3	14.3	11	13	0.355	4	1	R-13009-1	0	0	5	0	1	2.5	1	2	5	20	11	26	18
	**Range**	**85.0–86.0**	**76.0**	**1.12–1.13**	**19.3–19.8**	**11.0–11.8**	**18.3–21.7**	**13.8–14.3**						**Range**													
**Females (N = 5)**	R-13169	54.0	54.0	1.00	13	9	13.5	10.4	11	14	0.370	6	0	R-13169	1	0	5	1	2	2	0	4	6	20	9	27	19
	R-11913	70.0	67.0	1.05	18.2	10.9	17.4	13.0	9	12	0.358	5	1	R-11913	0	0	6	1	3	2	1	3	3	16	9	24	20
	R-13011-2	60.0	60.0	1.00	16.6	9.6	14.8	10.7	8	12	0.397	4	0	R-13011-2	0	0	5	0	2.5	1	1	3	7	17	8	25	18
	R-13009-2	60.0	60.0	1.00	15.5	9.3	15.0	13.3	10	15	0.333	5	0	R-13009-2	1	0	4.5	0	2.5	1	1	3	3	18	8	25	18
	R-12202	54.0	51.0	1.06	13.7	8.9	13.3	11.0	8	12	0.392	3	0	R-12202	1	0	5	0	2.5	2	1	4	7	18	7	26	18
	**Range**	**54.0–70.0**	**51.0–67.0**	**1.00–1.06**	**13.7–18.2**	**8.9–10.9**	**13.3–17.4**	**10.7–13.3**	**6–11**	**10–15**	**0.33-0.39**	**3–6**	-	**Range**	-	-	**4.5–6**	–	**1–3**	**1–2.5**	**0–2**	**2–4**	**3–7**	**16–20**	**7–11**	**24–27**	**18–21**
	**Mean ± S.D.**	**59.6±6.5**	**58.4±6.2**	**1.02±0.03**	**16.0±1.9**	**9.7±0.9**	**15.1±1.7**	**12.0±1.3**	**9.0±1.8**	**12.6±1.6**	**0.4±0.0**	**4.7±1.1**	-	**Mean ± S.D.**	-	-	**5.2±0.4**	-	**2.0±0.8**	**1.6±0.5**	**1.0±0.6**	**3.1±0.7**	**5.4±1.8**	**18.1±1.5**	**8.7±1.3**	**25.7±1.3**	**18.9±1.2**

### Taxonomy

MtDNA data strongly indicates the presence of two deeply divergent clades within *Ph. mystaceus*: one from northeastern Iran, the other occupying the rest of the species range in Middle Asia (see Fig. 1). MtDNA divergence in *COI* gene fragments between these lineages is significant, 6.84–7.28% of substitutions, what corresponds to species-level divergence values in lizards, including the genus *Phrynocephalus* (Nagy et al. 2012; Nazarov et al. 2012, 2014; Solovyeva et al. 2012, 2014; Hartmann et al. 2013; Nazarov & Poyarkov 2013; Amarasinghe et al. 2017; Orlova et al. 2017). According to the data of Solovyeva et al. (2014), sequences of three other mtDNA genes of Iranian and Middle-Asian lineages of *Ph. mystaceus* are also deeply divergent: ND4 (6.6%), ND2 (8.0%) and cyt b (6.6%). Divergence time estimates (Solovyeva et al., 2018) suggest that the split between Iranian and Middle Asian *Ph. mystaceus* happened in the Pliocene, ca. 3.7 Ma (6.0–2.0 Ma). Thus, our data strongly indicate the presence of a deep-divergent mtDNA lineage of *Ph. mystaceus* in northeastern Iran which deserves taxonomic recognition.

The question of the taxonomic status proposed for the Khorasan *Ph. mystaceus* populations, is, however, a matter of taste. On one hand, biogeographically the Khorasan *Ph. mystaceus* populations appear to be isolated from the main part of the species range in Middle Asia. The sands of the northeastern Iranian Plateau are located on much higher elevations (700-1000 m a.s.l.) than in the Middle Asia where *Ph. m. mystaceus* sensu lato occur (usually, 0-400 m a.s.l.), and are separated from the Caspian Basin by the Kopet-Dagh mountains, which has an estimated geologic uplift time of 5 Ma (Smit et al. 2013). The formation of Kopet-Dagh might be responsible for the initial split between the populations of *Ph. mystaceus*. The montane area of Kopet-Dagh lacking habitats suitable for *Ph. mystaceus*, such as sand dunes, serves as a barrier preventing gene flow between the Middle Asian and the Khorasan populations. Geographic isolation resulted in deep molecular divergence might suggest that the full species status should be proposed for the Khorasan populations of *Ph. mystaceus*.

However, despite the significant molecular divergence, morphological differentiation between the Khorasan and Middle Asian linages of *Ph. mystaceus* is weak with only few morphological characteristics separating them. At the same time, individuals of *Ph. mystaceus* from the vast range in the Caspian Region and Middle Asia are poorly differentiated both by morphometric characteristics (see Vel’dre 1964a, 1964b; Semenov and Shenbrot 1990; Golubev and Sattorov 1992) and by mtDNA (this paper). High morphological plasticity and variability are often recorded in specialized psammophilous groups of lizards (see Semenov and Shenbrot 1990). Both mtDNA and morphological data fail to resolve differentiation between the currently recognized non-Iranian subspecies of *Ph. mystaceus*: *mystaceus* sensu stricto, “*galli*” and “*aurantiacocaudatus*”. These subspecies are not supported as respective monophyletic groups in mtDNA analysis: the variation pattern is more likely clinal along the range from Xinjiang of China and Eastern Kazakhstan westwards to Middle Asia and Caspian Region. This suggests a recent dispersal of the non-Iranian *Ph. mystaceus* ancestor from a refugium in Eastern Kazakhstan westwards towards Caspian Basin.

There is no morphological or mtDNA evidence for recognizing *Ph. m. galli* as a distinct subspecies; we therefore confirm the conclusions of Semenov and Shenbrot (1990) who regarded *Ph. m. galli* as a junior synonym of the nominative subspecies. The East Kazakhstan *Ph. m. aurantiacocaudatus* is paraphyletic with respect to *Ph. m. mystaceus* and is not supported as a valid taxon according to our mtDNA data. The only existing character distinguishing *Ph. m. aurantiacocaudatus* from the representatives of other populations from Caspian Region and Middle Asia is the bright orange-red coloration of the tail ventral surface in juvenile specimens. Unfortunately, this character cannot be verified on museum collections since orange tail coloration fades quickly after preservation. Analysis of morphometric and meristic characters could separate *Ph. m. aurantiacocaudatus* from the nominative form *Ph. m. mystaceus*. We conclude that the subspecific status of *Ph. m.* “*aurantiacocaudatus*” requires further justification.

Our data show that the significant genetic differentiation of Khorasan *Ph. mystaceus* and presence of a number of stable diagnostic morphological characters warrants its recognition as a separate taxon. As noted above, genetic divergence between *Ph. mystaceus* from Khorasan and individuals from the rest of the species range is high, comparable or even exceeds the species-level genetic distances in *Phrynocephalus* (Solovyeva et al. 2014). However, we tentatively refrain from proposing the full species status for this lineage, and suggest that, at least at the current stage of research, it should be recognized as a subspecies, for the following three reasons:

(1) Due to matrilineal way of mtDNA inheritance and absence of recombination, even deep genetic divergence in mtDNA markers, does not guarantee reproductive isolation and should not serve as a sole reason for suggesting the full species status.

(2) Morphologically, the Khorasan population is still quite similar to other *Ph. mystaceus* populations and the revealed morphological differences are mostly quantitative, further morphological evidence is needed.

(3) Our sampling from Khorasan Province of Iran is limited, further studies in northeastern Iran are needed to uncover new populations in the area between the *Ph. m. mystaceus* range in Turkmenistan and the Khorasan population, genetic and morphological characterization of these populations is required.

A recent analysis had shown that subspecies are getting more rarely proposed for the extant reptiles in the last 50 years (Uetz and Stylianou 2018), what is connected with a growing tendency to elevation of many subspecies to species and also with growing prevalence of the phylogenetic species concept (Cracraft 1983), which does not recognize subspecies. However, we still consider subspecies to be a useful taxonomic category for reflecting geographic variation and evolutionary specificity in wide-ranged complexes of reptiles. Though taxonomic status of Middle Asian subspecies “*galli*” and “*aurantiacocaudatus*” is questionable, both mtDNA sequences and external morphology of the Khorasan population of *Ph. mystaceus* significantly differ from all other populations of this species. This allows us to describe it herein as a new subspecies:

#### Phrynocephalus mystaceus khorasanus ssp. n.

Taxon classificationAnimaliaSquamataAgamidae

http://zoobank.org/6E926506-3D7A-4C99-BF64-A02C48157B5C

**Figure 6.**
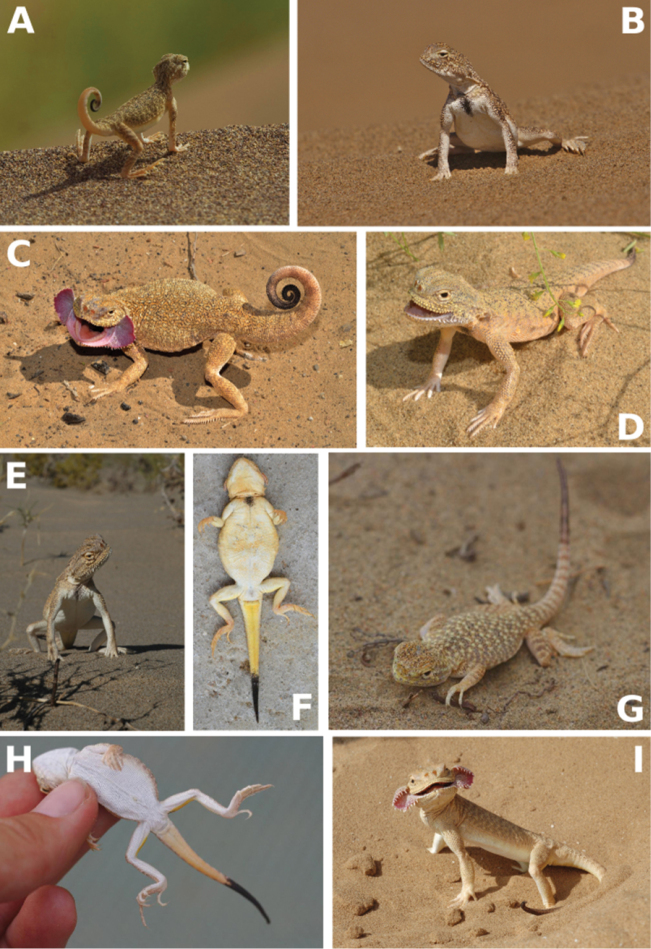
*Ph. mystaceus* in life: **A** subadult *Ph. mystaceus khorasanus* ssp. n., orange lower surface of the tail is shown, Iran (photograph by R. A. Nazarov) **B** *Ph. mystaceus khorasanus* ssp. n., female, Iran (photo by R. A. Nazarov) **C** *Ph. m. mystaceus*, Russia, Astrakhan region, Dosang (photograph by E. A. Dunayev) **D** *Ph. m. mystaceus*, Dagestan, Sarykum sands (photograph by E. A. Dunayev) **E** *Ph. m. mystaceus*, Uzbekistan, Qarakalpaqiston (corresponds to the previously recognized subspecies “*galli*”; photograph by E. A. Dunayev) **F** *Ph. m. mystaceus*, Dagestan, Sarykum sands (corresponds to the previously recognized subspecies “*dagestanica*”; photograph by E. A. Dunayev) **G** *Ph. m. aurantiacocaudatus*, E Kazakhstan, SE Balkash Lake (photograph by E. N. Solovyeva) **H** *Ph. m. aurantiacocaudatus*, E Kazakhstan, SE Balkash lake (photograph by E. N. Solovyeva) **I** *Ph. m. mystaceus*, Russia, Astrakhan region, Dosang (photograph by E. A. Dunayev).

**Figure 7.**
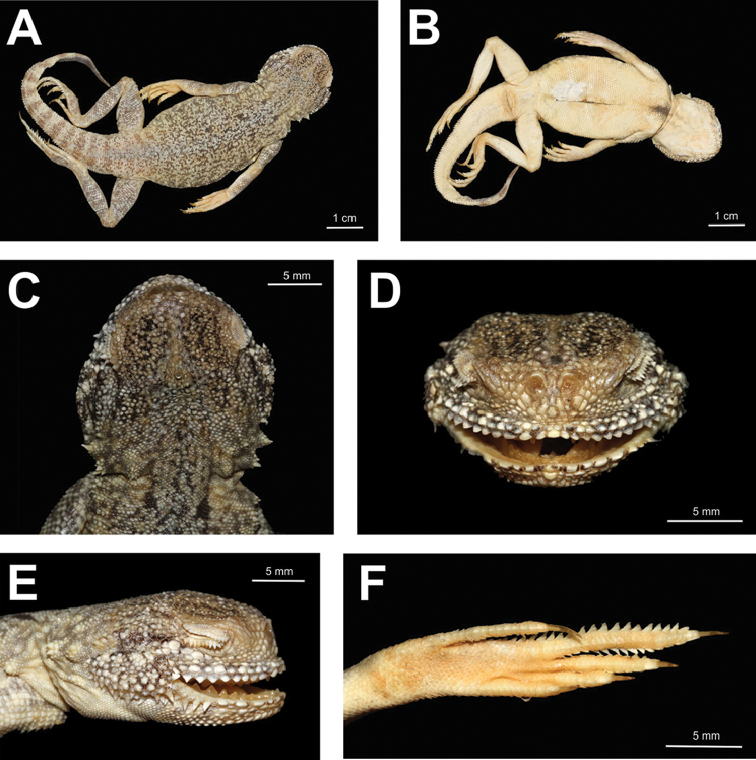
Holotype of *Ph. mystaceus khorasanus* ssp. n. in preservative: **A** dorsal view **B** ventral view **C** head in dorsal view **D** head in frontal view **E** head in lateral view; **F** right foot in thenar view (photographs by E. N. Solovyeva).

**Figure 8.**
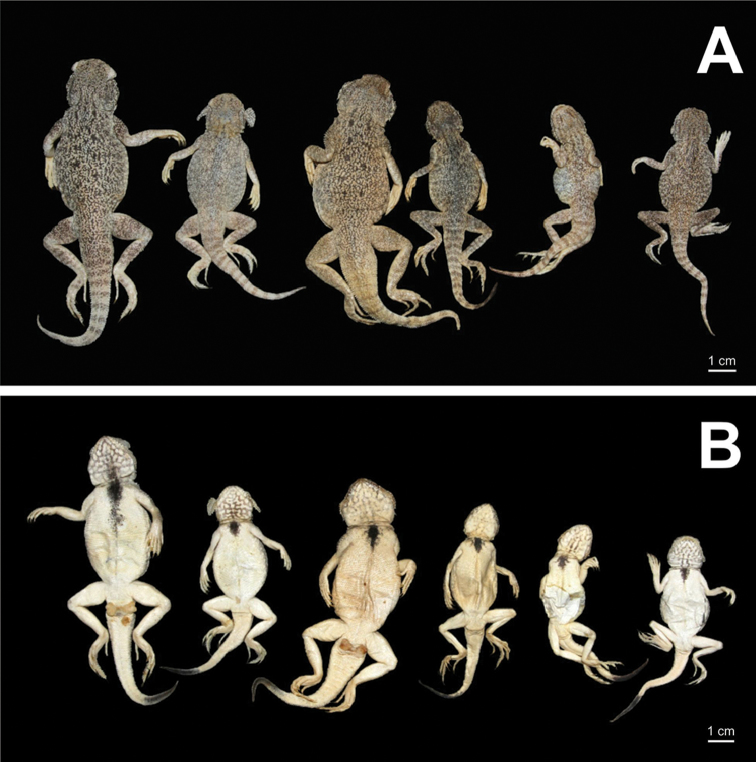
Paratypes of *Ph. mystaceus khorasanus* ssp. n. in preservative: **A** in dorsal view **B** in ventral view (photographs by E. N. Solovyeva).

Figs 6A, B ; 7 ; 8 ; Table 4

##### Holotype.

ZMMU R-11913 (adult female; field number NR-1191).

##### Type locality.

**Figure 9.**
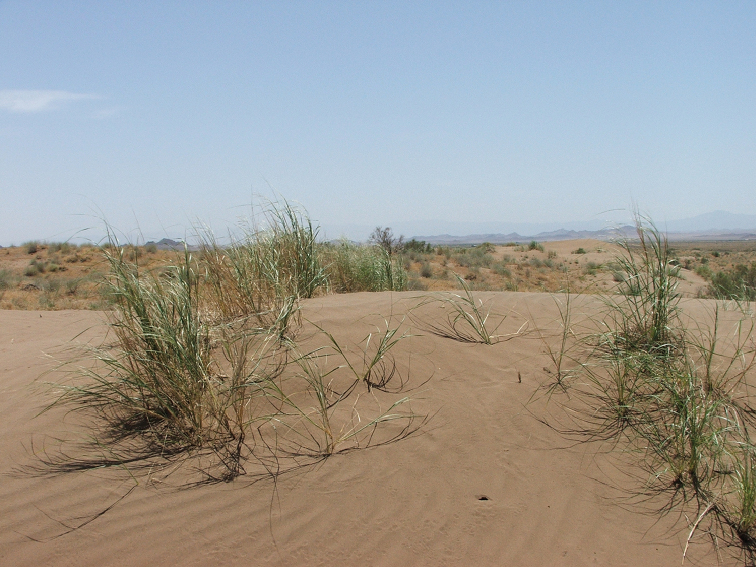
Typical habitat of *Ph. mystaceus khorasanus* ssp. n. at the type locality in the vicinity of Gonabad, Khorasan Razavi Province, Iran (photo by R. A. Nazarov).

Iran, Khorasan historical area, Khorasan Razavi Province (ostan), environs of Gonabad, the right bank of the Kale-Shur River; sand dunes (see Fig. 9); N34°39', E58°43'; elevation 850 m a. s. l. Collected by Roman A. Nazarov and Mehdi Radjabizadeh on April 25, 2005.

##### Paratypes.

ZMMU R-13009 (one adult male with everted hemipenial structures, field number RAN 1723; and one adult female, field number RAN 1724) was collected in Iran, Khorasan historical area, Khorasan Razavi Province, 20 km east of the town of Boshrouyeh (N33°54', E57°30'; elevation 864 m a. s. l.) by Dmitriy A. Bondarenko, Roman A. Nazarov, and Mehdi Radjabizadeh on May 05, 2009. The rest of paratypes were collected in the area close to the type locality. ZMMU R-13011 (one adult male with hemipenial structures, field number RAN 1947; and one subadult female, field number RAN 1948) was collected in Iran, Khorasan Razavi Province, 60 km north of the town of Gonabad, stabilized or semi-stabilized sands (N34°36', E58°14'; elevation 867 m a. s. l.) by Roman A. Nazarov, Rustam K. Berdiev, Vlad G. Starkov, and Mehdi Radjabizadeh on June 02, 2009. ZMMU R-13169 (subadult female) was collected in Iran, Khorasan Razavi Province, 30 km north of the town of Gonabad, on sandy massif on the right bank of the Kale-Shur river (N34°35', E58°43'; elevation 888 m a. s. l.) by Roman A. Nazarov, Dmitriy A. Bondarenko, and Mehdi Radjabizadeh on May 10, 2010. ZMMU R-12202 (juvenile female with slightly orange lower surface of the tail, field number N-093) was collected in Iran, Khorasan Razavi Province, 60 km north of the town of Gonabad, on sands (N34°36', E58°44'; elevation 881 m a.s.l.) by Dmitriy A. Bondarenko on April 20, 2006.

##### Diagnosis.

A member of *Ph. mystaceus* species complex based on the following combination of morphological attributes: (1) a large-sized *Phrynocephalus* species with SVL up to 97.5 mm, tail shorter than SVL; (2) pair of cutaneous flaps present at mouth corners with numerous spiny scales along flap edges; (3) distinctly flattened body and tail; (4) toes with fringes formed by triangular scales; subdigital lamellae on toes III and IV with ridges. *Phrynocephalus mystaceus khorasanus* ssp. n. can be distinguished from the nominative subspecies of *Ph. mystaceus* by the following combination of two diagnostic morphological characteristics: (1) 24–27 lamellae on toe IV; (2) few supralabial scales (less than 14). In life, the new subspecies can be further distinguished from the nominative subspecies by the orange color of the lower surface of tail in young specimens (lemon to yellowish in *Ph. m. mystaceus* except the populations from Eastern Kazakhstan and western China, formerly described as *Ph. m. aurantiacocaudatus*). MtDNA sequences of *Phrynocephalus mystaceus khorasanus* ssp. n. are markedly distinct from those in all other populations of *Ph. mystaceus* with sequence divergence in the range of 6.84–7.28% between them. The new subspecies is notably smaller than the representatives of southern populations of *Ph. m. mystaceus* from Uzbekistan and Turkmenistan, formerly described as *Ph. m. galli*, which can reach SVL up to 122.7 mm (Anderson 1999), whereas for Iranian population Anderson (1999) reported the largest specimen of *Ph. mystaceus* to have SVL up to 77.7 mm. SVL in the largest specimen in our sampling reached 86.0 mm, while Molavi et al. (2014) recorded a specimen with SVL of 97.5 from Semnan Province.

##### Etymology.

The name of the new subspecies khorasanus is a Latinized toponymic adjective, derived from Khorasan, the name of the historic area and a Khorasan Razavi Province in the northeast Iran, where the new subspecies was found. We suggest the “Khorasan Secret Toad-headed Agama” as a common name in English.

##### Description of holotype.

Medium-sized agamid lizard, adult female, specimen in good state of preservation; body dissected on ventral side along the midline of belly (dissection ca. 20 cm in length). Measurements and counts of the holotype are presented in Table 4.

Head large, rounded, distinctly wider than neck region (see Fig. 7A); body and tail notably flattened. Snout abruptly blunt, head almost vertical in profile view (see Fig. 7E), nostrils invisible dorsally (see Fig. 7C). Nasals separated from each other by single scale (see Fig. 7D). Dorsal surface of head with distinct pileus consisting of small slightly keeled scales; ca. 30 scales across the pileus. Pineal scale separated from nasals by 13 smaller scales; scales covering orbital area somewhat smaller than those on frontal surface of head; occipital scales not enlarged. Five scales contacting subnasal ventrally (see Fig. 7D). Subnasal scale not in contact with inner (medial) side of supranasal. Supralabials separated from subnasal scale by 6 rows of small granular scales (see Fig. 7D). Pair of skin-folds form characteristic ear-shaped flaps in mouth corners, edges of each flap with enlarged conical scales, two groups of similarly enlarged conical scales on each side of head posterior to the mouth angle at tympanal area (see Fig. 7E). Supralabial scales anterior to cutaneous fold at mouth angle 11/12 (hereafter data for symmetrical characteristics is given in Right/Left order); 9/9 of anterior supralabials notably flattened, 2/3 posterior supralabials conical-shaped; supralabials separated from small granular scales of lower eyelid by 3/4 rows of scales, ventral row of these scales almost the same size as supralabials (see Fig. 7E). Single small scale between the posteriormost supralabial and insertion of cutaneous fold at mouth angle. Infralabial scales anterior to cutaneous fold — 6/6, 3/3 of anterior infralabials notably flattened, posterior infralabials cone-shaped. Posterior corner of eye and insertion of cutaneous fold at mouth angle separated by row of three enlarged flat scales (see Fig. 7E). Vertebral scales not enlarged. Scales at middle of dorsum slightly bigger than scales on dorsolateral and lateral surfaces of body. Dorsal scales with weak keels, becoming cone-shaped laterally, forming almost triangular spines on the flanks. Notably enlarged spiny scale (about four times the size of adjacent scales) on each side of thorax behind maxilla, two groups of enlarged spiny scales on each lateral surface of neck region (see Fig. 7E). Tail notably flattened along its whole length. Scales on dorsal surface of tail and on ventral surface of tail posterior half notably keeled; scales on lateral sides of tail with well-pronounced spines. Limbs comparatively long: hindlimb length greater than distance from cloaca to gular fold. Toe IV bearing a single row of subdigital lamellae, each with a well-pronounced ridge on its volar surface; lateral sides of toe IV with two rows of enlarged triangular scales that form distinct serrated fringe (see Fig. 7F). Similar crests present on lateral surfaces of toe III, triangular scales on toe III notably smaller compared to those on toe IV (see Fig. 7F). Number of lamellae on toe IV 24/24, on toe III 16/16; number of enlarged triangular scales on toe IV 20/20, on toe III 9/9.

##### Color of holotype in life.

In life dorsum sandy-beige; with numerous small black and white dots and reticulations; row of three pairs of irregular-shaped larger dark blotches on each side of vertebral line; ventral surfaces of body, limbs and proximal part of tail white; ventral surface of tail tip black, chin and throat with gray reticulations, chest with blackish longitudinal blotch. Ten brownish transverse bars (wider than interspaces) on dorsal surface of tail, faint at tail basis, get more distinct towards tail tip. Internal surfaces of mouth angle cutaneous flaps in life are pinkish, and may become red when animal displays a threatening posture.

##### Color of holotype in preservative.

In preservative, numerous dark spots and mottling are distinct on dull sandy-gray background color of dorsum. They form vermiculate patterns ca. 1–2 scales wide. On lateral parts of dorsum these lines form 6–7 indistinct dark transverse bands. Ten dark transverse bars on dorsal side of tail are well-distinct (Fig. 7A). Three posterior dark bars have a distinct light-beige longitudinal line between them along midline of tail. Tail ventral surface light yellowish-white. Ventral surface of head with distinctive dark greyish marbling (Fig. 7A). Distinct triangular longitudinal black spot in the middle of chest area resembling a “necktie”, ca. 8.8 mm in length. Black coloration of distal part of ventral surface of tail 24 mm in length.

##### Paratype variation.

Variations of morphological characteristics in the type series are shown in Table 4 and in Fig. 8. In general, morphology of paratypes corresponds well to morphology of the holotype. SVL of new subspecies varies in range of 85.0–86.0 mm in two males, and in range of 54.0–70.0 in five females; tail length 76.0 mm in males, 51.0–67.0 mm in females; tail comparatively shorter in male specimens (SVL/TL ratio 1.12–1.13) than in females (SVL/TL ratio 1.00–1.06); however, the sample size is too small to detect significant differences. Length of dark distal part of ventral surface of tail varies from 20 to 27 mm. Number of subdigital lamellae on toe III varies from 17 to 20, from 25 to 28 on toe IV. Number of enlarged triangular scales of lateral fringes on toe III from 7 to 11, on toe IV from 18 to 21. Number of flattened anterior supralabials 6–11, total number of supralabials (to insertion of cutaneous fold at mouth angle) varies from 10 to 15. Number of small scales ventrally in contact with subnasal scale 3–6. Subnasal scale in all paratypes (except one specimen ZMMU R-13009) touches supranasal along medial edge of latter. In nearly all paratypes supralabials are separated from subnasal by five rows of small scales (only in ZMMU R-13009 by 4/5 rows of small scales). In most specimens, there is one small scale between last supralabial and insertion of cutaneous fold at mouth angle (specimen ZMMU R-13011 has two scales, ZMMU R-13169 lacks such scales). Number of flat anterior infralabials varies from 2 to 4, total number of infralabials to insertion of cutaneous fold at mouth angle varies from 5 to 7 (only ZMMU R-13009 has 3/3 infralabials). Number of black irregularly shaped spots on dorsum also may vary: from 4 to 6 pairs of black spots on each side of vertebral line (see Fig. 8A).

We were unable to detect sexual dimorphism in morphometric and meristic characteristics of *Ph. mystaceus khorasanus* ssp. n., however our sample size (N = 7) was too small. Molavi et al. (2014), who also examined seven specimens of both sexes from Semnan Province, was also unable to detect sexual dimorphism in morphological features in their sample.

##### Distribution.

To date, the new subspecies is known from two major localities in southwestern part of Khorasan Razavi Province (environs of the towns of Gonabad and Boshrouyeh, this study) and from a single locality in the easternmost part of Semnan Province of Iran (Ahmad Abad village, Molavi 2014). The record from the environs of the town of Boshrouyeh appears to be the southernmost known locality for *Ph. mystaceus* complex known to date. The three records of *Ph. mystaceus* by Anderson (1999) from the northern part of Khorasan Razavi Province, North Khorasan and Golestan provinces are all located along the border with Turkmenistan. These populations most likely correspond to *Ph. m. mystaceus* rather than to *Ph. mystaceus khorasanus* ssp. n. as they are close to the range of the nominative form and there are no biogeographic barriers that separate these populations. On the contrary, localities in Khorasan Razavi and Semnan provinces are situated on different elevations and sand massifs are isolated from the range of *Ph. m. mystaceus* by at least 200 km of habitats unsuitable for *Ph. mystaceus*. We anticipate new records of the new subspecies in sandy areas of Khorasan Razavi, Semnan and, possibly, northern part of Yazd and South Khorasan provinces.

##### Habitat.

*Ph. mystaceus khorasanus* ssp. n. inhabits sandy areas with sparse vegetation in northeast Iran at comparatively higher altitudes, than other *Ph. mystaceus* subspecies. The usual habitat is represented by dunes of loose sands and semi-stabilized dunes with rare grass, occasional bushes of *Haloxylon* sp. and *Tamarix* sp. and large open sandy areas (Fig. 9). The areas inhabited by the new subspecies receive almost no rainfall during the year. In the town of Gonabad the average annual temperature is 17.3 °C, the average temperature in July reaches 29.2 °C, the average temperature in January is 4.8 °C; In Boshrouyeh the average annual temperature is 19.7 °C, the average temperature in July is 31.9 °C, the average temperature in January is 6.6 °C. (http://www.climate-data.org).

Lizards burrow in sand, digging short tunnels and chambers; they can quickly dig into sand by rapid lateral movements of the body (Anderson, 1999).

##### Comparisons with other subspecies.

Comparisons of the new subspecies from Khorasan Razavi and Semnan provinces of Iran with the nominative subspecies *Ph. m. mystaceus* sensu lato from Middle Asia, Caspian basin, and westernmost Xinjiang (China) are summarized below. In preservative, the new subspecies can be differentiated from specimens of *Ph. m. mystaceus* by the following combination of morphological attributes: lower number of subdigital lamellae on the IVth toe (SLIV 25.7 (24–27; N = 7) in *vs.* 30.2 (25–35; N = 70) in *Ph. m. mystaceus* sensu lato); comparatively lower number of supralabials (SL 12.1 (10–14; N = 7) *vs.* 14.9 (10-19; N = 70) in *Ph. m. mystaceus* sensu lato) and by the comparatively shorter black distal part on the tail ventral surface (TL-black/TL 0.38 (0.36–0.40; N = 7) *vs.* 0.42 (0.32–0.48; N = 70) in *Ph. m. mystaceus* sensu lato). In life, juvenile and young specimens of the new subspecies can be further distinguished from Middle Asian / Caspian Basin populations of *Ph. mystaceus* by is rusty orange color of the proximal part of tail ventral surface (vs. lemon-yellow in *Ph. m. mystaceus* sensu stricto), but is similar to orange tail coloration in juveniles of East Kazakhstan – western China populations described as *Ph. m. aurantiacocaudatus*.

We do not recognize *Ph. m. galli* as a separate subspecies due to the absence of stable genetic and morphological differences of this subspecies from *Ph. m. mystaceus* (see above). The *Phrynocephalus mystaceus dagestanica* form from Daghestan (Ananjeva “1986” 1987) is very close to the populations from the Volga River basin and was considered a synonym of *Ph. m. mystaceus* by several authors (Semenov and Shenbrot 1990; Barabanov and Ananjeva 2007). Our molecular and morphometric data do not support monophyly or significant differentiation of *Ph. m. aurantiacocaudatus* from Eastern Kazakhstan and western China. The only stable difference between this population and *Ph. m. mystaceus* sensu stricto is the tail coloration in juveniles. We consider that additional genetic and morphological data is needed to clarify taxonomic status of East Kazakhstan *Ph. mystaceus* populations.

##### Discussion.

Our study indicates deep genetic divergence between Iranian populations of *Ph. m. khorasanus* ssp. n. and the rest of the populations within the range of the species. However, morphological differentiation within *Ph. mystaceus* complex is less clear with only a few morphological characteristics that reliably separate these two lineages. Differentiation pattern for the mtDNA *COI* gene within the Middle Asian and Caspian populations of *Ph. mystaceus* complex suggests that East Kazakhstan was populated by *Ph. mystaceus* earlier than the rest of Middle Asia. After that, a dispersal process from the east to the west likely took place. Morphologically different populations of *Ph. mystaceus* across Middle Asia present considerable amount of variation both in body size and in such morphological features as the relative size of cutaneous flaps in the mouth angles, relative tail length, etc. This high morphological plasticity may be connected with psammophilous biology of this species, as it was suggested by previous researchers (Vel’dre 1964a, 1964b; Semenov and Shenbrot 1990; Golubev and Sattorov 1992).

The data of phylogenetic analyses in the present paper clearly indicates that the whole territory of Middle Asia, including westernmost China and Caspian region, is inhabited by a single poorly differentiated mtDNA lineage. Golubev and Sattorov (1992) argued that coloration of the ventral tail surface in juveniles of *Ph. mystaceus* is also subject to high variation, and “orange-” and “yellow-tailed” specimens can be occasionally recorded within the same population, thus suggesting that subspecies within *Ph. mystaceus* should not be recognized. Our mtDNA genealogy indicates that both *Ph. m.* “*galli*” and *Ph. m.* “*aurantiacocaudatus*” do not form a respective monophyletic units and are genetically indistinguishable or very close the nominative subspecies *P. m. mystaceus* sensu stricto (p-distance 1.65–1.87% in case of East Kazakhstan populations).

On the contrary, the Khorasan population described herein as *Ph. m. khorasanus* shows very deep genetic divergence in mtDNA which is comparable to the species-level divergence in *Phrynocephalus*, but is only moderately differentiated morphologically. Indeed, previous research on four mtDNA genes also showed significant differentiation between *Ph. mystaceus* from Khorasan and *Ph. m. mystaceus* (*p*-distances: *COI* – 7.18%; *ND4* – 6.6%; *ND2* – 8.0%; and *cyt b* – 6.6%) (see Solovyeva et al. 2014). According to our unpublished data on molecular dating of 4 mtDNA genes these two forms diverged during Pliocene about 3.7 Ma (Solovyeva et al., 2018). Further studies are required to verify the taxonomic status of *Ph. m. khorasanus* ssp. n., including morphological examination of larger samples and molecular analysis of the nuclear DNA markers in order to check the presence of possible isolation between the Iranian and Middle Asian forms of *Ph. mystaceus*. The new subspecies inhabits sand dunes in the northeastern Iran; this desert area is separated from the range of *Ph. m. mystaceus* by Kopet-Dagh Mountain Ridge making the possibility of gene flow between these populations quite low. However, the taxonomic status of *Ph. mystaceus* populations reported by Anderson (1999) from northern Iran (northern parts of Golestan, North Khorasan and Khorasan Razavi provinces) is unclear and require verification. Additional fieldwork in northern Iran, western Afghanistan, and southern Middle Asia is required to recover new populations of *Ph. mystaceus* complex. Further progress in understanding of the phylogenetic relationships within *Ph. mystaceus* complex might lead to reconsideration of the taxonomic status of the Khorasan population as a full species.

**Figure 10.**
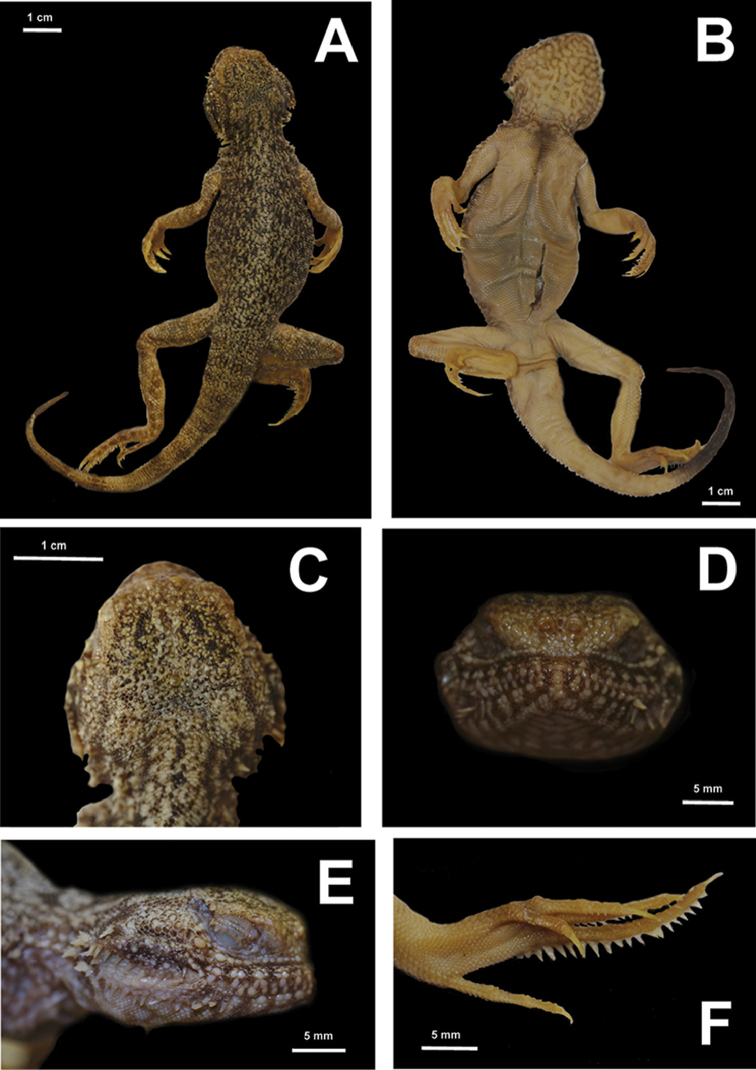
ZMMU R-6413, lectotype of *Phrynocepahlus mystaceus galli* Krassowsky, 1932 in preservative: **A** dorsal view **B** ventral view **C** head in dorsal view **D** head in frontal view **E** head in lateral view **F** left foot in thenar view (photographs by E. N. Solovyeva).

**Figure 11.**
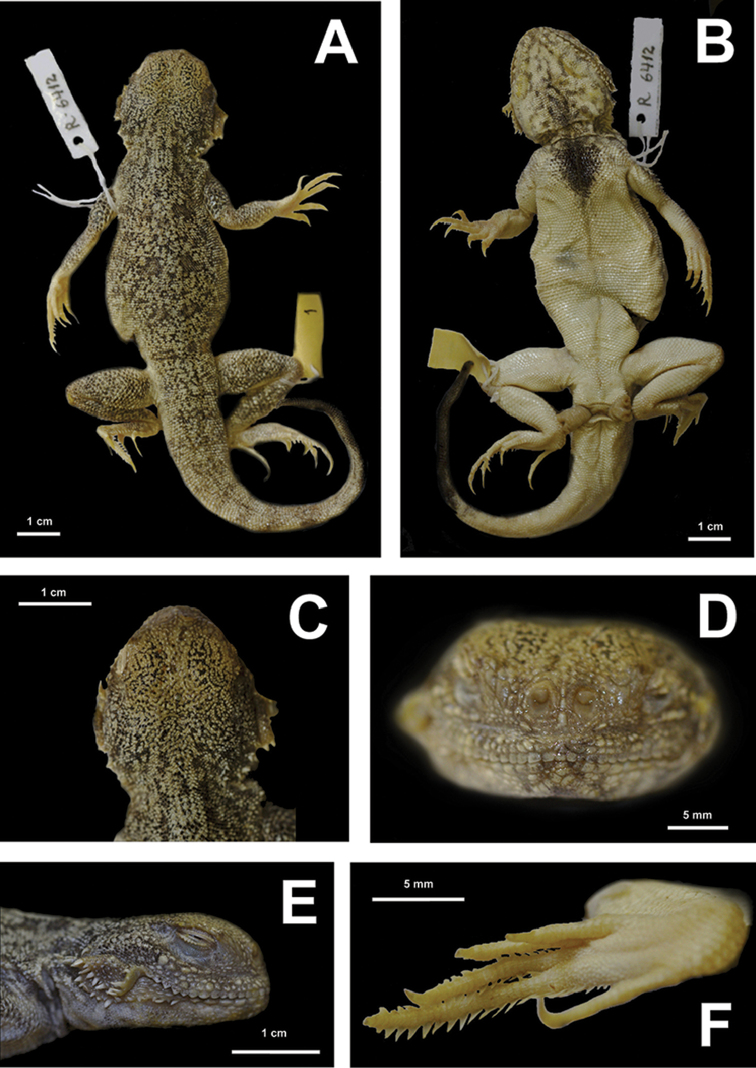
ZMMU R-6412, holotype of *Phrynocepahlus mystaceus aurantiacocaudatus* Semenov & Shenbrot, 1990 in preservative: **A** dorsal view **B** ventral view **C** head in dorsal view **D** head in frontal view **E** head in lateral view **F** right foot in thenar view (photographs by E. N. Solovyeva).

## Supplementary Material

XML Treatment for Phrynocephalus mystaceus khorasanus

## Appendix 1

**Material examined in morphological analysis.**

***Phrynocephalus mystaceus mystaceus***: Kalmykia (ZMMU R-3455 [N = 8: 4 females, 4 males]); Russia, Astrakhan, Dosang (ZMMU R-8696 [N = 12: 9 females, 3 males]). Additionally, we examined the holotype ZMMU R-6412.

***Phrynocephalus mystaceus galli***: Turkmenistan, Repetek (ZMMU R-2043 [N =11: 6 females, 5 males], ZMMU R-2045 [N =9: 5 females, 4 males]). Additionally, we examined the lectotype ZMMU R-6413; **lectotype** of *Phrynocepahlus mystaceus galli* Krassowsky, 1932 (ZMMU R-6413, previously part of ZMMU R-2047; male, Turkmenistan, Repetek; coll. on 07-09.08.1929 by S.S. Turov, L.G. Turova; see Fig. 10).

***Phrynocephalus mystaceus aurantiacocaudatus***: Kazakystan, Muyunkum sands (ZMMU R-6858 [N = 4: 2 females, 2 males], ZMMU R-6566 [N = 1 female]); Kazakhstan, Ili River (ZMMU R-3794 [N = 7: 4 females, 3 males]); Kazakhstan, Alma-Aty (ZMMU R-10906 [N = 1 juvenile]); Kazakhstan, left bank of Ili River (ZMMU R-12518 [N = 1 female]); Kazakhstan, Kapchagay (ZMMU R-12140 [N = 2 juveniles]); Kazakhstan (ZMMU R-2051 [N = 3: 1 female, 1 male, 1 juvenile)]; Kazakhstan, Bakanas (ZMMU R-7470 [N = 2 females]); Kazakhstan, right bank of Ili river (ZMMU R-2828 [N = 4: 2 females, 1 male, 1 juvenile]); Kazakhstan, Djarkent env. (ZMMU R-2049 [N = 5: 2 females, 1 male, 2 juveniles]); Kazakhstan, SE Balkhash env. (ZMMU R-557 [N = 2: 1 female, 1 subadult]); **holotype** of *Phrynocepahlus mystaceus aurantiacocaudatus* Semenov & Shenbrot, 1990 (ZMMU R-6412; male, East Kazakhstan, 70 km N-N-W from Ushtobe, 45°50'N; 77°40'E; coll. on 12-13.06.1987 by D.V. Semenov, G.I. Shenbrot; see Fig. 11).

***Phrynocephalus mystaceus khorasanus* ssp. n.**: Iran, Khorasan (ZMMU R-11913 [N = 1 female], ZMMU R-13011 [N = 2: 1 female, 1 male], ZMMU R-12202 [N = 1 female], ZMMU R-13169 [N = 1 female], ZMMU R-13009 [N = 2: 1 female, 1 male]).

## Appendix 2

**Table A2.** Mann-Whitney test of independent series: “aura” – *Ph. m. “aurantiacocaudatus*”, “ir” – *Ph. m. khorasanus* ssp. n., “myst” – *Ph. m. mystaceus* sensu stricto, “galli” – *Ph. m. “galli*”; for abbreviations, see Materials and Methods. Significant values of p ≤ 0.05 are marked with bold and an asterisk.

	ALL							Sexes
	aura-ir	ir-myst	ir-galli	myst-aura	aura-galli	galli-myst	ir-all	f-m
**1. SVL**	0.321	**0.000***	**0.013***	**0.000***	**0.008***	**0.000***	**0.005***	**0.007***
**2. TL**	**0.044***	**0.000***	**0.001***	**0.000***	**0.027***	**0.000***	**0.000***	**0.005***
**3. SVL/TL**	**0.008***	**0.000***	0.115	**0.001***	**0.006***	**0.000***	**0.001***	0.591
**4. SLbA**	**0.015***	**0.004***	**0.031***	0.178	0.855	0.180	**0.014***	0.813
**5. SL**	**0.007***	**0.001***	**0.050***	**0.003***	0.455	**0.005***	**0.003***	0.355
**6. TL-black/TL**	**0.023***	**0.000***	**0.001***	**0.002***	**0.016***	0.499	**0.001***	0.527
**7. SSbNb**	0.351	0.623	**0.036***	0.412	**0.037***	**0.003***	0.140	0.201
**8. SbN-SpN**	0.596	0.597	0.158	0.050	**0.000***	0.118	0.570	0.169
**9. SpN**	0.927	0.923	0.103	**0.000***	**0.002***	**0.009***	0.457	**0.022***
**10. hSpN SbN**	**0.015***	**0.000***	**0.033***	0.127	0.758	0.096	**0.001***	0.259
**11. SbN-L**	**0.010***	0.589	0.097	**0.000***	0.992	**0.016***	0.144	0.234
**12. WS&BL**	0.111	**0.000***	**0.027***	0.026	0.026	NA	**0.002***	0.524
**13. aIMd-IL**	**0.017***	0.392	0.053	0.113	0.771	0.252	**0.030***	0.091
**14. SuSSCF**	0.029	**0.004***	0.912	0.164	**0.000***	**0.000***	0.174	0.839
**15. pSL-CF**	0.272	**0.026***	**0.004***	**0.042***	**0.000***	**0.025***	**0.007***	0.857
**16. ILbA**	**0.006***	**0.046***	**0.002***	**0.022***	0.510	**0.002***	**0.003***	0.640
**17. IL**	**0.023***	**0.017***	0.059	0.645	**0.013***	0.059	**0.022***	0.588
**18. SLIII**	**0.009***	**0.011***	**0.008***	**0.011***	0.738	**0.002***	**0.001***	NA
**19. FrIII**	0.341	**0.000***	0.056	**0.000***	**0.000***	0.889	0.283	
**20. SLIV**	**0.000***	0.087	**0.000***	0.087	0.463	**0.026***	**0.000***	
**21. FrIV**	**0.001***	**0.000***	0.937	**0.000***	**0.000***	**0.026***	0.563	

**Table A2 (Continued).**

	MALES							FEMALES						
	aura-ir	ir-myst	ir-galli	myst-aura	aura-galli	galli-myst	ir-all	aura-ir	ir-myst	ir-galli	myst-aura	aura-galli	galli-myst	ir-all
**1. SVL**	0.432	0.142	1.000	**0.004***	0.533	**0.002***	0.110	0.147	**0.001***	**0.003***	**0.000***	**0.007***	**0.001***	**0.003***
**2. TL**	0.694	**0.034***	0.160	**0.002***	0.328	**0.000***	0.726	**0.015***	**0.001***	**0.003***	**0.000***	0.052	**0.000***	**0.001***
**3. SVL/TL**	0.260	**0.033***	**0.040***	**0.047***	0.947	**0.009***	**0.022***	**0.005***	**0.002***	0.921	**0.006***	**0.000***	**0.000***	**0.045***
**4. SLbA**	0.279	**0.028***	0.550	0.057	0.215	**0.003***	0.281	**0.039***	0.086	**0.028***	0.861	0.547	0.837	**0.040***
**5. SL**	**0.043***	**0.032***	0.293	**0.037***	0.063	**0.005***	**0.043***	**0.048***	**0.009***	**0.045***	**0.038***	0.771	0.101	**0.023***
**6. TL-black/TL**	0.091	**0.033***	**0.040***	0.395	0.141	0.186	**0.032***	0.186	**0.004***	**0.048***	**0.002***	0.263	0.077	**0.018***
**7. SSbNb**	0.715	1.000	0.197	0.450	0.089	**0.010***	0.537	0.457	0.601	0.106	0.752	0.148	0.073	0.230
**8. SbN-SpN**	0.330	0.892	0.593	0.178	**0.017***	0.296	0.892	0.804	0.526	0.125	0.150	**0.007***	0.229	0.453
**9. SpN**	0.436	0.361	0.091	0.663	**0.040***	0.143	0.192	0.901	0.844	0.109	0.932	0.034	**0.037***	0.928
**10. hSpN SbN**	0.103	0.049	0.176	0.441	0.813	0.392	0.053	0.063	**0.003***	**0.041***	0.174	0.899	0.156	**0.006***
**11. SbN-L**	0.125	0.797	0.223	**0.040***	0.662	0.102	0.349	**0.043***	0.582	0.243	**0.002***	0.851	0.066	0.314
**12. WS&BL**	0.192	**0.034***	0.061	0.279	0.338	NA	0.118	0.231	**0.006***	**0.003***	0.045	**0.030***	NA	**0.011***
**13. aIMd-IL**	0.528	0.454	0.294	0.520	0.466	1.000	0.611	**0.002***	0.130	**0.007***	0.097	0.818	0.208	**0.004***
**14. SuSSCF**	0.608	0.310	0.648	0.482	0.092	**0.030***	1.000	**0.021***	**0.008***	0.913	0.221	**0.000***	**0.000***	0.090
**15 pSL-CF**	0.641	0.892	0.355	0.283	**0.032***	0.154	0.643	0.099	**0.021***	**0.003***	0.110	**0.002***	0.119	**0.004***
**16. ILbA**	**0.037***	0.054	**0.049***	0.261	0.207	0.070	**0.026***	0.091	0.351	**0.039***	**0.039***	0.966	**0.016***	**0.036***
**17. IL**	0.480	0.633	0.227	0.710	0.216	0.154	0.354	**0.031***	**0.012***	**0.010***	0.354	**0.038***	0.261	**0.034***
